# Metabolic Alterations of Short-Chain Organic Acids in the Elderly Link Antibiotic Exposure with the Risk for Depression

**DOI:** 10.3390/metabo14120689

**Published:** 2024-12-07

**Authors:** Shujing Sun, Li Kong, Fangting Hu, Sheng Wang, Menglong Geng, Hongjuan Cao, Xingyong Tao, Fangbiao Tao, Kaiyong Liu

**Affiliations:** 1 School of Public Health, Anhui Medical University, No. 81 Meishan Road, Hefei 230032, China; ssj9680@163.com (S.S.); kongli821367106@163.com (L.K.); 17856927546@163.com (F.H.); gengmengl@ahmu.edu.cn (M.G.); txyayd@126.com (X.T.); fbtao@ahmu.edu.cn (F.T.); 2 Center for Big Data and Population Health of IHM, Anhui Medical University, No. 81 Meishan Road, Hefei 230032, China; 3 Center for Scientific Research, Anhui Medical University, No. 81 Meishan Road, Hefei 230032, China; wangsheng@ahmu.edu.cn; 4 Key Laboratory of Population Health Across Life Cycle, Ministry of Education of the People’s Republic of China, No. 81 Meishan Road, Hefei 230032, China; 5 Anhui Provincial Key Laboratory of Environment and Population Health Across the Life Course, No. 81 Meishan Road, Hefei 230032, China; 6 Lu’an Center of Disease Control and Prevention, Lu’an 237000, China; chj@lacdc.com.cn

**Keywords:** antibiotics, biomonitoring, depressive symptoms, short-chain organic acids, the elderly

## Abstract

Background: Our previous study showed that antibiotic exposure was linked to depressive symptomatology in community-dwelling older adults in China. Our current study aims to explore the underlying mechanisms by assessing the intermediated effects of circulating short-chain organic acids (SCOAs) on this association. Methods: Depressive symptoms were screened by the 30-item Geriatric Depression Scale (GDS-30). Urinary concentrations of antibiotics and serum SCOAs were measured using a liquid chromatography–mass spectrometry method. Results: Increased exposure to sulfadiazine, azithromycin, tetracyclines, or veterinary antibiotics (VAs) was positively associated with GDS-30 scores. Tetracycline reduced levels of caproic acid, iso-butyric acid, and iso-caproic acid (iso-CA), with iso-CA concentration inversely correlating with GDS-30 scores, while β-hydroxybutyric acids showed a positive correlation. The mediating effect of serum iso-CA on the association between depression and ofloxacin, with a mediating effect of 25.3%, and the association between depression and tetracycline, with a mediating effect of 46.3%, were both statistically significant, indicating partial mediation. Conclusions: Antibiotics may affect the levels of SCOAs in older adults and could potentially contribute to depressive symptoms by influencing alterations in serum iso-CA levels.

## 1. Introduction

The elderly population is growing rapidly in low- and middle-income countries, while the mental health issues of this population are generally underemphasized, and over 90 percent of cases of major depressive disorder are untreated in these countries [1]. Specifically, in China, the prevalence of depressive symptoms among older adults has ranged from 33.8% in 2015 to a staggering 50.6% in 2018, indicating a significant burden of depressive symptoms [2]. Beyond all doubt, the disease burden of depressive symptoms in China has been on the rise and will continue to increase in the coming years. The etiology of depression is complex. Researchers have identified some risk factors of depressive symptoms including genetic, social, and environmental influences [3,4].

In the last 10 years, increasing evidence has revealed that the gut microbiota has an enormous impact on the development of depression through the gut–brain axis [5]. Environmental pollutants, such as residual antibiotics as emerging contaminants, can influence the gut microbial composition, indicating a high association with the increased incidence of depression [6,7,8]. Also, several lines of studies have suggested the close correlation between antibiotic exposure from clinic use and food-chain residues and the risk of developing depression [9,10]. Although the etiology of depression is still not clear, clinical studies and animal experiments have demonstrated that exposure to antibiotics can lead to disruptions in the gut microbiota [11,12].

It is well known that antibiotics, even at environmentally relevant concentrations, can directly or indirectly cause dysbiosis in the gut microbiota [13], and this gut dysbiosis is marked by substantial changes in the abundance of gut microbiota and the levels of its metabolites such as classical short-chain fatty acids (SCFAs) in circulatory systems [14,15,16,17]. These alterations of SCFAs might link cumulative antibiotic exposure with the risk of depression and could help unveil its pathological mechanisms [18,19]. Notably, short-chain organic acids (SCOAs) affect brain function by regulating mitochondrial bioactivities [20,21]. Also, SCFAs are included in SCOAs. SCOAs can be categorized based on the number of carbon atoms [22]. Most SCOAs are produced through the microbial fermentation of dietary fibers and other indigestible carbohydrates in the gut. This fermentation process is carried out by specific gut microbes, including *Firmicutes* and *Bacteroidetes*, which break down complex carbohydrates into simpler compounds, such as SCFAs. These compounds are essential intracellular metabolites [23,24,25]. In addition, some microbes can produce SCOAs through the β-oxidation of fatty acids [26]. The production of SCOAs is primarily regulated by the associated microbes, including *Firmicutes*, *Lachnospiraceae*, *Ruminococcaceae*, *Bacteroidaceae*, and *Proteobacteria*, through changes in their relative abundance [14]. Specifically, *Escherichia coli*, a model strain in industry, is usually used to produce various SCOAs [23,27]. In addition to the classical SCFAs, it is crucial to investigate the relationship between other SCOAs and depression. Specifically, attention should be directed towards substances like lactic acid (LA), β-hydroxybutyrate (BHB), and crotonic acid in understanding their potential connection with depression. An animal study revealed that LA’s antidepressant effect primarily stems from reducing histone deacetylase 5 (HDAC5) levels and enhancing histone H3 acetylation in the hippocampus of depressed mice [28]. Moreover, BHB, a metabolic byproduct of fatty acids, has demonstrated antidepressant effects [29,30]. Studies have identified significant modification of the H3K56 site in the histone modification locus by SCOAs (e.g., crotonic acid, BHB), indicating their crucial roles in epigenetic regulation [31]. Additionally, the biological impacts of environmental factors on depression and other psychiatric stress-related disorders are mediated through a range of epigenetic modifications.

In light of this, our objective was to pioneer the investigation into the correlation among urinary biomonitoring of antibiotics, serum SCOA levels, and depression. Furthermore, we sought to validate the hypothesis that common SCOAs intermediates play a mediating role in the association between antibiotic exposure and depression. This study offers novel insights into the impact of antibiotic pollutants and specific serum SCOAs on depression among the elderly, aiding in a comprehensive understanding of the risks associated with antibiotic residues, even at very low doses, and potentially informing clinical approaches to treating depression.

## 2. Methods and Materials

### 2.1. Study Population and Design

**Figure 1:**
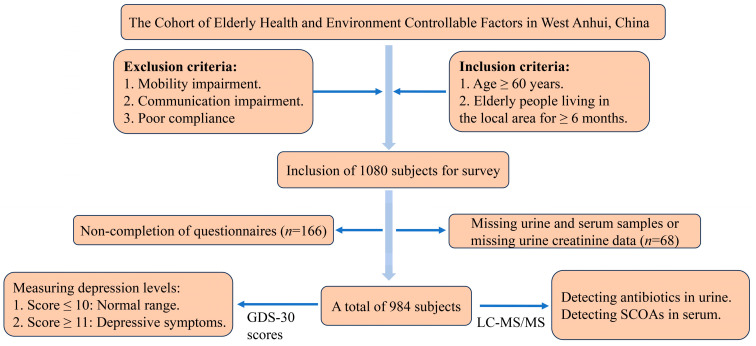
A graphical representation of the overall work plan. GDS-30 scores, 30-item Geriatric Depression Scale scores; LC-MS/MS, liquid chromatography–tandem mass spectrometry; SCOAs, short-chain organic acids.

The participants were from the Cohort of Elderly Health and Environment Controllable Factors in West Anhui, China (Figure 1). Briefly, a multistage stratified random sampling method was used to select 1080 older adults. We excluded the elderly with missing urine and serum samples or missing urine creatinine data (*n* = 68) and non-completion of questionnaires (*n* = 166). Individuals with severe physical or mental diseases or who had used antibiotics in the preceding month were excluded. Ultimately, the analysis included data from 984 participants aged 60–94 years. Venous blood samples and urine samples were collected from the participants after an overnight fast and stored at −80 °C for subsequent detection. A structured questionnaire was used by trained staff and graduate students at local hospitals to collect information on demographic characteristics, lifestyle, disease history, activities of daily living (ADL), and cognitive function. The 30-item Geriatric Depression Scale (GDS-30) was administered to assess the presence of depressive symptoms, with a total score of 30 points, including 10 reverse-scoring items and 20 positive-scoring items. In this study, a score of ≥11 was considered indicative of the presence of depressive symptoms [32]. All participants provided informed consent before the investigation. This study was reviewed and approved by the Human Research Committee of Anhui Medical University (ethical clearance number for the population study: 20170284; Approval date: 1 March 2016).

### 2.2. Detection of Urinary Antibiotics

We employed liquid chromatography–tandem mass spectrometry (LC-MS/MS) to screen 45 antibiotics and 2 antibiotic metabolites in urine samples. The experimental procedures, methodological validity, urine creatinine determination, and use of urine creatinine adjustments of antibiotic levels were thoroughly documented in our previous publication [33,34]. Briefly, the thawed urine samples were centrifuged at 4 °C for 10 min at 4000 rpm. Following centrifugation, 1 mL of the supernatant was transferred to a 4 mL brown glass bottle, to which 200 μL of McIlvaine-Na_2_EDTA buffer (pH 4.0), 20 μL of antibiotic internal standard mix, and 15 μL of β-glucosidase were sequentially added. The sealed bottles were vortexed for 1 min and incubated in a constant-temperature air bath shaker at 37 °C and 100 rpm for 10 h to facilitate enzyme digestion. After digestion, the sample was transferred to an activated solid-phase extraction (SPE) column, from which it was naturally eluted following the SPE process. The column we used was a ZORBAX SB-C18 (150 mm × 2.10 mm, 3.50 μm, Agilent, Santa Clara, CA, USA). Sample detection was performed using an LC-MS/MS system (1200 HPLC-6410 MS, Agilent, Santa Clara, CA, USA), employing positive ion mode for electrospray ionization and negative ion mode in Dynamic Multiple Reaction Monitoring (DMRM) for sample analysis. The mass spectrometric parameters, including ionization mode, retention time, and the quantitative ion pair for detecting antibiotics and their internal standards, have been previously described in detail [34].

### 2.3. Measurement of Short-Chain Organic Acids in Serum

The accurate detection and quantification of short- and medium-chain fatty acids in a total of 14 different human biofluids by reversed-phase liquid chromatography (RPLC) followed by tandem mass spectrometry (MS/MS) has been investigated [35]. In the present study, according to the published articles, a total of 11 SCOAs (Table S1) were detected. The detailed method and procedure are outlined in our previous study [36]. In brief, the thawed serum samples (60 μL) were mixed with 500 ng/mL internal standard (10 μL) and cold acetonitrile (120 μL), and the resultant mixture was centrifuged. Subsequently, 2 mol/L 3-nitrophenylhydrazine hydrochloride (3-NPH)•HCl) (5 μL) and 1.2 mol/L 1-(3-Dimethylaminopropyl)-3-ethylcarbodiimide hydrochloride (EDC•HCl) (30 μL) were added to the supernatant (60 μL) for derivatization. After the reaction was stopped, the solution was dried at room temperature under nitrogen. After resolution and centrifugation, the supernatant (85 μL) was separated using the Dionex Ultimate 3000 ultrahigh-performance liquid chromatograph (Thermo Fisher; Waltham, MA, USA) system equipped with a Infinity Lab Proshell 120 EC-C18 column (2.1 mm × 100 mm, 2.7 μm, Agilent, Santa Clara, CA, USA) and analyzed using the Q-Exactive plus quadrupole-electrostatic field orbital trap high-resolution mass spectrometer (Thermo Fisher; Waltham, MA, USA).

### 2.4. Statistical Analysis

Demographic characteristics, such as age, sex, marital status, education level, and solitary living arrangements, have been associated with depression. Considering the effect of lifestyle on depression and serum SCOAs levels in the elderly, we incorporated variables such as drinking habits, physical activity, and dietary structure into our analytical model.

After logarithmically transforming the corrected antibiotic concentrations, we conducted multivariate linear regression analyses to evaluate the dose–response relationships. These analyses specifically explored the associations between urinary antibiotics and GDS-30 scores, between antibiotics and serum SCOAs, and between SCOAs and GDS-30 scores, respectively. Furthermore, we attempted a mediation analysis to investigate if SCOAs might mediate the relationship between antibiotic exposure and depression. The criteria employed to define a mediator were as follows: (a) a substantial effect of changes in urinary antibiotic levels on the concentration of the mediating SCOAs and (b) a significant correlation between the presence of the mediating SCOAs and the occurrence of depression.

All statistical analyses were conducted using SPSS, version 23.0 (SPSS, Chicago, IL, USA), and R software (version 4.1.0; Vienna, Austria). A significance level of α = 0.05 was selected for 2-sided tests.

## 3. Results

### 3.1. Baseline Characteristics

Table 1 provides an overview of the baseline characteristics of the participants and of those exhibiting depressive symptoms. According to the questionnaire survey, 447 (45.4%) the elderly were male, and 500 (50.9%) were aged >70 years. Data from 984 patients were included in the analyses. In total, 27.7% of the participants exhibited depressive symptoms. A chi-square test revealed that sex, marital status, physical activity, education level, living alone, drinking, dietary structure, and cognitive function were associated with depression.

### 3.2. Association Between Antibiotic Use and Depression

Antibiotics with a detection rate of ≥10% in the entire sample underwent individual analysis [33,37,38]. In all, 12 antibiotics, namely sulfaclozine, trimethoprim, penicillin V, azithromycin, ofloxacin, ciprofloxacin, norfloxacin, enrofloxacin, tetracycline, oxytetracycline, doxycycline, and florfenicol, were detected (detection rate ≥ 10%). Considering the distribution characteristics of antibiotic residues in the older population, as reported in our previous study, concentrations below the limits of detection (LODs) were adjusted to 1/2 LODs. Detected antibiotics with different mechanisms of action were then grouped into six categories: sulfonamides, β-lactams, macrolides, fluoroquinolones, tetracyclines, and chloramphenicol. Furthermore, antibiotics were classified based on their intended usage into human antibiotics (HAs), veterinary antibiotics (VAs), antibiotics preferred as HAs (PHAs), and antibiotics preferred as VAs (PVAs). The antibiotic concentrations were normalized by urinary creatinine concentration and are expressed as micrograms per gram of creatinine (μg/g of creatinine).

The antibiotic with the highest detection rate (35.5%) was sulfaclozine. The class of antibiotics with the highest detection rate (55.7%) was sulfonamides (Tables S2 and S3). Following adjustments of all aforementioned covariates (Table S4), the per-unit increment of sulfaclozine was positively correlated with GDS-30 score (*β* = 0.456, 95% CI: 0.034, 0.878), and exposure to VAs was also positively correlated with GDS-30 score (*β* = 0.666, 95% CI: 0.002, 0.329).

Similarly, in the high-exposure group of 984 elderly individuals in Model 2, sulfaclozine (OR = 1.557, 95% CI: 1.059, 2.291), azithromycin (OR = 1.701, 95% CI: 1.022, 2.831), and VAs (OR = 1.477, 95% CI: 1.028, 2.123) were associated with an increased risk of depression (Tables S5 and S6). The possible nonlinear dose–response relationship between antibiotic exposure and depression was assessed using a restricted cubic spline (RCS) analysis. The results revealed that an increase in sulfaclozine or VAs was associated with an increased risk of depression, although the association between VAs and depression was not significant (Figures S1 and S2).

### 3.3. Short-Chain Organic Acids Levels

The distribution of individual SCOAs exhibited a positive and skewed distribution (Figure S3). The percentage of the total population with crotonic acid concentrations above the LOD of 0.5 ng/mL was 67.9%. Therefore, values below the LOD were replaced with 1/2 LOD (Table S7). With the exception of crotonic acid, the detection rate for all 10 SCOAs was 100%. Among the SCOAs, LA had the highest concentration (geometric mean: 3.68 µmol/L), and iso-caproic acid (iso-CA) had the lowest concentration (geometric mean: 0.88 nmol/L).

#### 3.3.1. Antibiotic Exposure and Short-Chain Organic Acids

**Figure 2:**
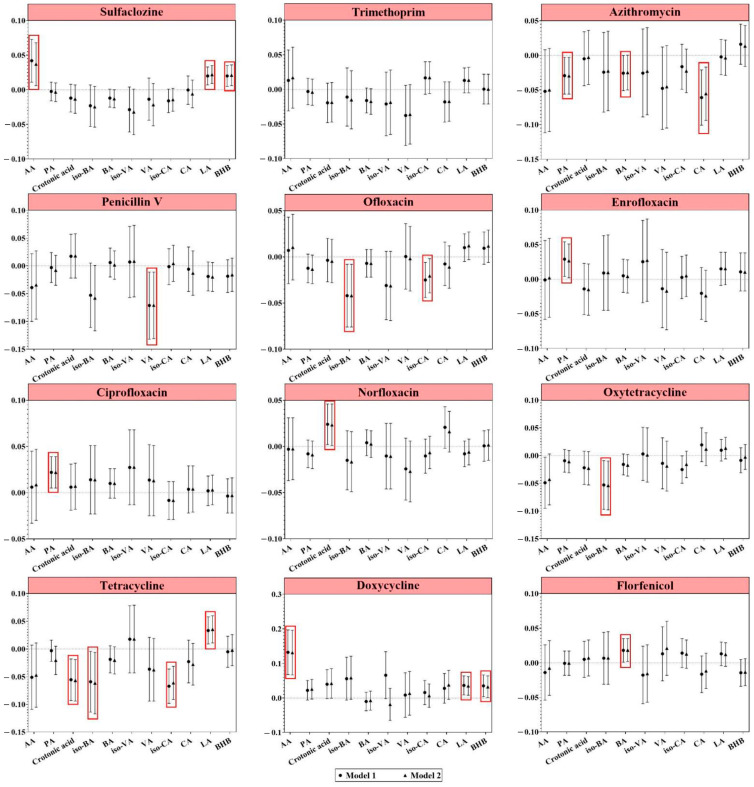
Associations of individual antibiotic exposure with serum short-chain organic acids. Model 1 was adjusted for age and gender. Model 2 was adjusted for age, gender, living alone, educational level, marital status, physical activity, drinking, cognitive impairment, and dietary structure by multiple linear regression. Abbreviation: AA, acetic acid; PA, propionic acid; BA, butyric acid; VA, valeric acid; CA, caproic acid; LA, lactic acid; BHB, β-hydroxybutyric acid. Red boxes: *p* < 0.05, indicating statistical significance.

Figure 2 illustrates the association between urinary antibiotic levels and serum SCOAs in our study cohort. Regarding specific antibiotics, increased exposure to sulfaclozine or doxycycline was associated with increased serum levels of acetic acid (AA), LA, and BHB. Additionally, urinary concentrations of enrofloxacin and ciprofloxacin exhibited a significant positive association with propionic acid (PA), with a 0.035-fold (95% CI: 0.011, 0.060) and 0.018-fold (95% CI: 0.002, 0.035) increase, respectively. By contrast, a significant inverse correlation was observed between ofloxacin exposure and iso-butyric acid (iso-BA) and iso-CA concentrations. Furthermore, each unit increase in urinary log-transformed tetracycline concentrations resulted in decreases in log-transformed caproic acid (CA), iso-BA, and iso-CA levels by 0.058-fold (95% CI: 0.017, 0.098), 0.061-fold (95% CI: 0.006, 0.117), and 0.061-fold (95% CI: 0.031, 0.091), respectively (Table S8).

As shown in Tables S9–S11, for specific antibiotics, exposure to sulfonamides was associated with increased levels of AA, LA, and BHB. In terms of the level of log-transformed BHB, there was a pattern for each unit increase in the urinary concentration of log-transformed VAs. By contrast, urinary concentrations of tetracyclines exhibited a significant inverse correlation with iso-CA (*β* = −0.029, 95%CI: −0.054, −0.055). Urinary PVA levels were significantly and negatively correlated with iso-CA concentrations. Bacteriostatic antibiotics were significantly and negatively correlated with iso-valeric acid (iso-VA) concentrations, and broad-spectrum antibiotics were also negatively correlated with iso-BA concentrations.

As shown, increased exposure to sulfaclozine, doxycycline, or sulfonamides may increase serum levels of AA, LA, and BHB. Exposure VAs may also increase the level of BHB. And, exposures to ofloxacin or tetracycline may both can decrease the level of the serum iso-BA and iso-CA. Additionally, exposure to tetracyclines or PVA is negatively correlated with the level of iso-CA.

#### 3.3.2. Association of Short-Chain Organic Acids with Depression

**Figure 3:**
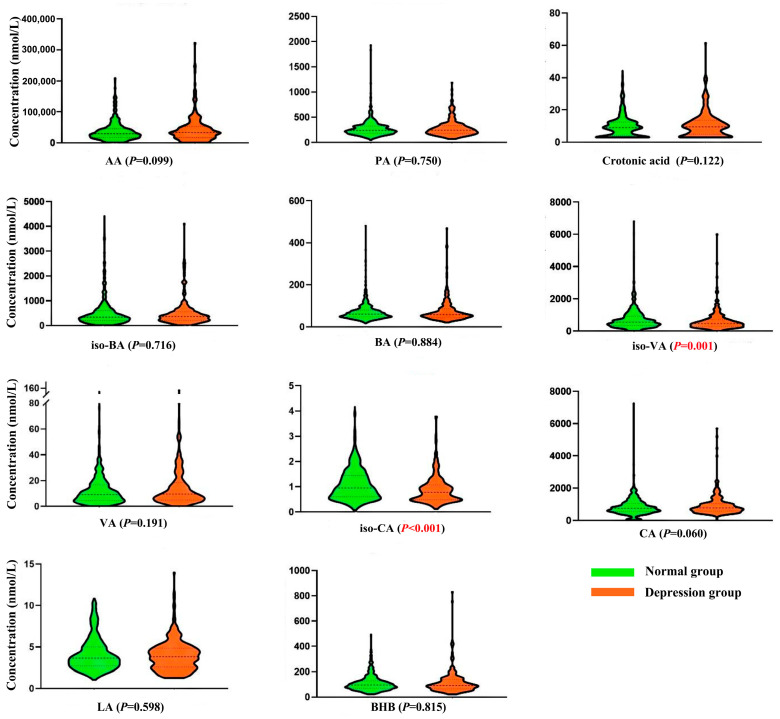
Distribution of short-chain organic acids in the elderly between normal and depression groups. AA, acetic acid; PA, propionic acid; BA, butyric acid; VA, valeric acid; CA, caproic acid; LA, lactic acid; BHB, β-hydroxybutyric acid. Red color, emphasizing *p* < 0.05, statistically significant.

In the univariate analysis, the Mann–Whitney test revealed higher levels of serum iso-VA and iso-CA concentrations in the older adult group with depression compared with the normal group (without depression) (Figure 3). Following the adjustment of variables in the multiple linear regression analysis (Table 2), each unit increase in the log-transformed iso-CA level led to a decrease in GDS-30 score (*β* = −1.758, 95% CI: −3.266, −0.250). Conversely, each unit increase in the log-transformed BHB level resulted in an increase in the GDS-30 score (*β* = 1.812, 95% CI: 0.190, 3.434). Furthermore, the results of the RCS analysis (Figure S4) revealed a significant nonlinear positive association between serum CA concentration and the risk of depression. As for GDS-30 scores, the result revealed that a higher level of iso-CA may decrease the GDS-30 score, but a higher level of BHB resulted in an increase in it.

#### 3.3.3. Mediating Effect of Short-Chain Organic Acids

**Figure 4:**
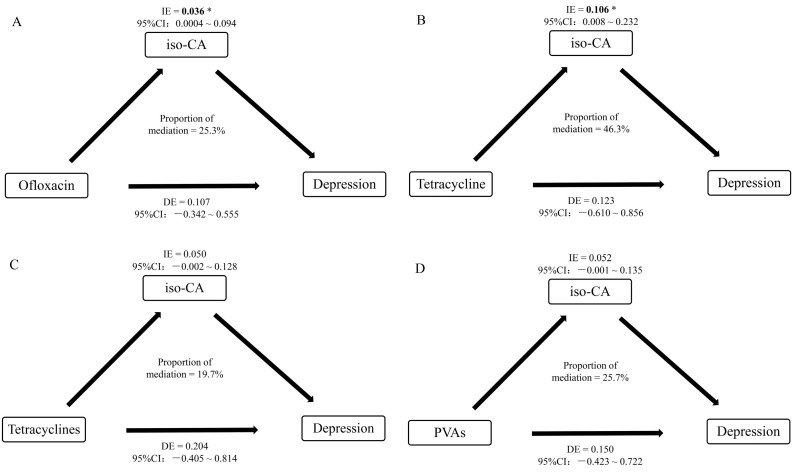
Intermediation of iso-CA. Adjusted for age, gender, living alone, educational level, marital status, physical activity, drinking, cognitive impairment, and dietary structure. (**A**) Mediating effect of serum iso-CA on the association between depression and ofloxacin; (**B**) Mediating effect of serum iso-CA on the association between depression and tetracycline; (**C**) Mediating effect of serum iso-CA on the association between depression and tetracyclines; (**D**) Mediating effect of serum iso-CA on the association between depression and PVAs. PVAs, antibiotics preferred as veterinary antibiotics; iso-CA, iso-caproic acid; IE, indirect effect; DE, direct effect; CI, confidence interval. * Bolding indicates *p* value < 0.05, statistically significant.

In the compiled analysis of associations between urinary antibiotic levels, GDS-30 score, and serum concentrations of SCOAs, serum BHB level (Figure S5) was found to positively mediate the association between depressive symptom score and sulfaclozine, sulfonamide, and VA levels in the elderly, with mediating effects of 8.6%, 27.2%, and 7.5%, respectively. However, these mediating effects were not statistically significant. Similarly, the mediating effect of serum iso-CA (Figure 4) on the association between depression, tetracyclines, and PVAs was not statistically significant. However, the mediating effect of serum iso-CA on the association between depression and ofloxacin, with a mediating effect of 25.3%, and the association between depression and tetracycline, with a mediating effect of 46.3%, were both statistically significant, which indicates partial mediation.

## 4. Discussion

We observed that specific antibiotic exposure among the elderly was associated with an increased risk of depressive symptoms. Specific organic acids, such as BHB and iso-CA, were identified to be associated with urinary antibiotic concentrations and GDS-30 score. The mediation analysis further indicated that iso-CA exhibited mediating effects on the relationship between antibiotic exposure and GDS-30 score among the elderly.

Recently, a Swedish cohort study on patients with cancer revealed that antibiotic use was associated with an increase in the risk of depression in three models adjusted for different confounders [39]. Lurie et al. [40] conducted three case–control studies between 1995 and 2013 that involved the use of a large UK population-based medical record database and revealed that the risk of depression was 1.24 (95% CI: 1.17, 1.30) or 1.31 (95% CI: 1.22, 1.41) times higher in patients using 1 course of sulfonamide antibiotics and 2–5 courses of sulfonamide antibiotics, respectively, compared with those not using sulfonamide antibiotics. Furthermore, a clinical study on fluoroquinolones reported that 62% of 94 fluoroquinolone users had depression [41]. However, these studies assessed antibiotic exposures by reviewing records of antibiotic prescription or self-reported medication. In our biomonitoring dada on antibiotic exposures, even after adjustment for relevant covariates, sulfaclozine and VAs were significantly associated with elevated risks of depression in the elderly, and a positive association was observed between the concentration of urinary fluoroquinolone and GDS-30 score [10].

As individuals enter old age, the previously stable intestinal flora undergo a transition to a more sensitive state and become highly susceptible to external influences [42]. Numerous studies have demonstrated that antibiotic exposure could disrupt the homeostasis of intestinal flora, resulting in alterations in the abundance of SCFA-producing bacteria, consequently affecting SCFA concentrations in the intestine [15,17]. As shown in our result that exposure to ofloxacin or tetracycline can decrease the level of serum iso-BA and iso-CA, exposure to tetracyclines or PVAs is negatively correlated with the level of iso-CA. In a randomized controlled trial involving 66 adults in the United States, exposure to amoxicillin/clavulanate for 7 consecutive days resulted in a gradual decrease in fecal concentrations of AA, PA, and butyric acid (BA). However, within a week after discontinuing the antibiotics, these concentrations gradually returned to pretreatment levels [16]. In our study, exposure to sulfaclozine, doxycycline, or sulfonamides may increase serum levels of AA. And, significant inverse correlations were observed between azithromycin exposure and PA, BA, and AA. Inconsistent with findings that the antibiotics neomycin and polymyxin B significantly increase iso-BA levels during the treatment of ischemic stroke [43], our results reveal that exposure to ofloxacin or tetracycline may decrease the level of serum iso-BA. The difference may be due to differences in the classes of antibiotics.

PA and BA are widely known as endogenous histone deacetylase (HDAC) inhibitors, directly suppressing the expression of HDAC and promoting histone hyperacetylation [44]. HDAC is implicated in brain development and associated with various neuropsychiatric disorders, including depression, schizophrenia, and Alzheimer’s disease [45]. Furthermore, BHB can also inhibit HDAC function, exert neuroprotective effects, and enhance brain-derived neurotrophic factor (BDNF) expression [46,47,48]. Exogenous BHB supplementation was demonstrated to alleviate reductions in BDNF expression and reduce depressive symptoms [49]. One study showed that mice with higher blood BHB levels tend to exhibit increased depression- and anxiety-like behaviors, suggesting endogenous BHB levels may be elevated in response to neuroinflammation or may be reflective of depressive behavior [50]. In our results, BHB was significantly and positively correlated with GDS-30 score, indicating that BHB may induce depression by disrupting energy metabolism in the population. Additionally, another study demonstrated that the concentration of iso-CA was significantly high in patients with depressive symptoms [51]. However, the results of our study showed that an increase in the log-transformed iso-CA level led to a decrease in the GDS-30 score. The inconsistent result is most likely due to the fact that our study assessed both male and female populations in general, and we used GDS-30 scores instead of Beck’s Depression Inventory scores.

Additionally, relevant studies have found that antibiotic intervention can lead to changes in the levels of gut microbiota metabolites such as AA, PA, and BA, along with subsequent mental and emotional disorders [52]. We speculate that the disruption of gut microbiota homeostasis and alterations in metabolite levels may be the underlying mechanisms by which antibiotics increase the risk of depression. In our study, exposure to ofloxacin, tetracycline, tetracyclines, or PVAs was significantly inversely associated with iso-CA concentration. The mediation effects of iso-CA between ofloxacin or tetracycline and depressive symptoms in the elderly were both significant. It has been theorized that iso-CA, produced by microorganisms, may undergo metabolism by host medium-chain acyl-coenzyme A dehydrogenase (MCAD) [53]. Through MCAD, the short chain of iso-CA, when coupled with glycine, generates the product isobutyrylglycine, which can co-elute with butyrylglycine. Conversely, in the absence of MCAD, the product isocaproylglycine accumulates. Their findings revealed that iso-CA supplied by Clostridium perfringens in the intestine increased isocaproylglycine levels in MCAD-deficient mice. From this, we can speculate that the pathway of microbial-produced isocaproic acid action in the human body may involve metabolism by MCAD and subsequent exertion in a different form. The iso-CA is also a reduced pathway product of leucine metabolism [53]. It has been shown that high-fat diet-associated intestinal flora release abundant leucine, which increases serum leucine levels, activates the mammalian target of rapamycin complex signaling pathway in myeloid progenitor cells, and promotes polymorphonuclear myeloid-derived suppressor cell differentiation, which promotes cancer progression. All the mentioned results indicated that antibiotic feeding was found to reduce tumor growth in both high-fat diet and high-fat diet fecal microbiota-transplanted mice [54]. Herein, we can hypothesize that antibiotics can affect serum leucine levels and thus iso-CA levels by defectively altering the state of the intestinal flora. And, there has been no evidence of a direct association between ofloxacin, tetracycline, and iso-CA, respectively. So, subsequent studies can further explore the potential role of iso-CA as a mediator in the influence of antibiotic exposure on late-life depression.

To the best of our knowledge, there has not been an epidemiological study investigating the role of SCOAs in the association between antibiotic exposure and depression among the elderly. Our research employed a biological monitoring method to evaluate antibiotic exposure levels in this natural population, offering a more accurate assessment compared to relying solely on prescription records. However, as the present study was a cross-sectional study only, it did not involve mechanistic explorations. And, we were unable to definitively establish a causal relationship between antibiotic exposure and SCOAs levels and their correlation with late-life depression symptoms. Finally, the irresponsible use of veterinary drugs, driven by a lack of scientific knowledge and a blind pursuit of economic gains by livestock producers, as well as the use of VAs in food animals, can result in the presence of drug residues in animal-derived food products such as meat, milk, and eggs, posing a potential threat to human health [55,56,57]. In the present study, however, the residues in the animal products of interest were not tested, and only a simple dietary composition was adjusted as a covariate, which represents one of the limitations of our study. Future studies should utilize longitudinal biological samples to monitor long-term antibiotic exposure and collect fecal samples for analyzing the role of the microbiota and its metabolites.

## 5. Conclusions

Exposure to elevated concentrations of sulfadiazine and VAs, respectively, heightened the risk of depressive symptoms in the elderly. Higher levels of BHB were linked to an increased risk of depression. The serum iso-CA concentration exhibited an inverse correlation with the GDS-30 score. Remarkably, serum iso-CA may mediate the association between exposure to ofloxacin and tetracycline and depression.

## Figures and Tables

**Table 1:** General characteristics of 984 subjects and their relation to depression.

Characteristics	*n* (%)	Depression	*p*-Value ^a^
		*n* (%)	
Gender			
Male	447 (45.4)	104 (23.3)	0.004
Female	537 (54.6)	169 (31.5)	
Age			
60~70	484 (49.1)	133 (27.5)	0.855
>70	500 (50.9)	140 (28.0)	
Marital status			
Non-widowed	256 (26.0)	100 (39.1)	<0.001
Widowed	728 (74.0)	173 (23.8)	
Physical activity			
Yes	292 (29.7)	43 (14.7)	<0.001
No	692 (70.3)	230 (33.2)	
Educational level			
Illiteracy	450 (45.7)	179 (39.8)	<0.001
Primary school	236 (23.9)	55 (23.3)	
Middle school	169 (17.2)	27 (16.0)	
High school	129 (13.1)	12 (9.3)	
Living alone			
Yes	134 (13.6)	62 (46.3)	<0.001
No	850 (86.4)	211 (24.9)	
Smoke			
Yes	189 (19.2)	46 (24.3)	0.278
No	795 (80.8)	227 (28.6)	
Drinking			
Yes	370 (37.6)	83 (22.4)	0.004
No	614 (62.4)	190 (30.9)	
Dietary structure			
Vegetable-based	551 (56.0)	172 (31.2)	0.009
Balanced	376 (38.2)	92 (24.7)	
Meat-based	57 (5.8)	9 (15.8)	
Salt			
Low	465 (47.3)	134 (28.8)	0.763
General	228 (23.1)	62 (27.2)	
High	291 (29.6)	77 (26.5)	
Oil			
Low	450 (45.7)	133 (29.6)	0.227
General	322 (32.8)	78 (24.2)	
High	212 (21.5)	62 (29.2)	
Sugar			
Low	617 (62.6)	178 (28.8)	0.428
General	256 (26.2)	64 (24.8)	
High	111 (11.3)	32 (28.8)	
Chronic diseases			
Yes	362 (36.8)	101 (27.9)	0.933
No	622 (40.0)	172 (27.7)	
Cognitive impairment			
Yes	407 (41.4)	62 (15.2)	<0.001
No	577 (58.6)	211 (36.6)	
Depression			
Yes	273 (27.7)	-	-
No	711 (72.3)	-	

^a^ Chi-square test. *p* < 0.05, statistically significant. BMI, Body Mass Index; ADL, Activities of Daily Living.

**Table 2:** Linear association between serum short-chain organic acids and GDS-30 score in the elderly.

Metabolites	Model 1 ^a^		Model 2 ^a^	
	*β* (95% CI)	*p*-Value	*β* (95% CI)	*p*-Value
AA	0.230 (−0.624, 1.083)	0.598	0.395 (−0.394, 1.184)	0.326
PA	1.013 (−0.933, 2.959)	0.307	0.581 (−1.228, 2.390)	0.529
Crotonic acid	0.380 (−0.845, 1.606)	0.543	0.056 (−1.074, 1.185)	0.923
iso-BA	−0.058 (−0.952, 0.836)	0.899	−0.150 (−0.974, 0.674)	0.721
BA	1.042 (−0.986, 3.070)	0.314	0.491 (−1.389, 2.370)	0.609
iso-VA	−0.370 (−1.187, 0.447)	0.374	−0.430 (−1.181, 0.321)	0.262
VA	0.529 (−0.333, 1.391)	0.229	0.266 (−0.539, 1.071)	0.516
iso-CA	−4.075 (−5.649, −2.501)	<0.001	−1.758 (−3.266, −0.250)	0.022
CA	2.470 (1.184, 3.756)	<0.001	1.024 (−0.200, 2.249)	0.101
LA	−0.916 (−2.944, 1.112)	0.376	0.316 (−1.562, 2.194)	0.742
BHB	0.769 (−0.986, 2.525)	0.390	1.812 (0.190, 3.434)	0.029

Note: GDS−30, Geriatric Depression Scale-30; AA, acetic acid; PA, propionic acid; BA, butyric acid; VA, valeric acid; CA, caproic acid; LA, lactic acid; BHB, β-hydroxybutyric acid; β, correlation coefficient; CI, confidence interval. Model 1 was adjusted for age and gender; Model 2 was adjusted for age, gender, living alone, educational level, marital status, physical activity, drinking, cognitive impairment, and dietary structure. ^a^ Model was analyzed by multiple linear regression. *p* < 0.05, statistically significant.

## Data Availability

The raw data supporting the conclusions of this article will be made available by the authors on request.

## Supplementary Materials

The following supporting information can be downloaded at: https://www.mdpi.com/article/10.3390/metabo14120689/s1. Figure S1: Nonlinear association of individual antibiotic exposure with depressive symptoms; Figure S2: Nonlinear associations between exposure to different types of antibiotics and depressive symptoms; Figure S3: Distribution of short-chain organic acids in the elderly; Figure S4: Nonlinear association between serum short-chain organic acids and risk of depression in the elderly; Figure S5: Intermediation of BHB. Table S1: The physicochemical information of short-chain organic acids; Table S2: Detection frequency and urinary concentration (μg/g) of individual antibiotics; Table S3: Detection frequency and urinary concentration (μg/g) of various antibiotics; Table S4: Associations between per lg10 increment of antibiotic concentrations and GDS-30 (*n* = 984); Table S5: Associations of creatinine-adjusted urinary individual antibiotics with depression in the elderly by multinomial logistic regression (less than LODs was used as the control) (*n* = 984); Table S6: Associations of creatinine-adjusted urinary various antibiotics with depression in the elderly by multinomial logistic regression (less than LODs was used as the control) (*n* = 984); Table S7: Distribution of short chain organic acids in serum; Table S8: Association between urinary single antibiotic levels and serum short chain organic acids in the elderly; Table S9: Association between urinary levels of various antibiotics (based on chemical structure) and serum short-chain organic acids in the elderly; Table S10: Association of use-based levels of various antibiotics in urine with serum short-chain organic acids in the elderly; Table S11: Association between urinary antibiotic levels (based on antimicrobial profiles and bactericidal mechanisms) and serum short-chain organic acids in the elderly.
