# Supplementary material for: Metabolic Alterations of Short-Chain Organic Acids in the Elderly Link Antibiotic Exposure with the Risk for Depression
Source: Metabolites. 2024 Dec 7;14(12):689. doi: 10.3390/metabo14120689 (PMC11679812; doi:10.3390/metabo14120689)
Supplement: Supplementary file 1 [file metabolites-14-00689-s001.zip › metabolites-3308700-supplementary.pdf]

Supplementary Material

Metabolic alterations of short-chain organic acids in the elderly link antibiotic exposures with the risk for depression

Shujing Sun[a, 1], Li Kong[a, 1], Fangting Hu[a], Sheng Wang[b], Menglong Geng[a, c, d], Hongjuan Cao[e], Xingyong Tao[a], Fangbiao Tao[a, c, d], Kaiyong Liu[a, c,d] *

[a] School of Public Health, Anhui Medical University, No. 81 Meishan Road, Hefei 230032, Anhui, China /Center for Big Data and Population Health, Institute of Health and Medicine, Hefei Comprehensive National Science Center; No 81 Meishan Road, Hefei 230032, Anhui, China

[b] Center for Scientific Research, Anhui Medical University, No. 81 Meishan Road, Hefei 230032, Anhui, China

[c] Key Laboratory of Population Health Across Life Cycle, Ministry of Education of the People's Republic of China, No. 81 Meishan Road, Hefei 230032, Anhui, China

[d] Anhui Provincial Key Laboratory of Environment and Population Health Across the Life Course

[e] Lu'an Center of Disease Control and Prevention, Lu'an 237000, Anhui, China

[1] These authors have contributed equally to this work.

* Correspondence to: School of Public Health, Anhui Medical University, No. 81 Meishan Road, Hefei, 230032, Anhui, China. Tel: +86 13696512858. E-mail: liukaiyong163@163.com. ORCID: 0000-0002-4492-9474.

Supplementary-Tables

Table S1. The physicochemical information of short-chain organic acids.

Table S2. Detection frequency and urinary concentration (μg/g) of individual antibiotics.

Table S3. Detection frequency and urinary concentration (μg/g) of various antibiotics.

Table S4. Associations between per lg10 increment of antibiotic concentrations and GDS-30 (*n* = 984).

Table S5. Associations of creatinine-adjusted urinary individual antibiotics with depression in the elderly by multinomial logistic regression (less than LODs was used as the control) (*n* = 984).

Table S6. Associations of creatinine-adjusted urinary various antibiotics with depression in the elderly by multinomial logistic regression (less than LODs was used as the control) (n = 984).

Table S7. Distribution of short chain organic acids in serum.

Table S8. Association between urinary single antibiotic levels and serum short chain organic acids in the elderly.

Table S9. Association between urinary levels of various antibiotics (based on chemical structure) and serum short-chain organic acids in the elderly**.**

Table S10. Association of use-based levels of various antibiotics in urine with serum short-chain organic acids in the elderly.

Table S11. Association between urinary antibiotic levels (based on antimicrobial profiles and bactericidal mechanisms) and serum short-chain organic acids in the elderly.

Supplementary- Figures

Figure S1 Nonlinear association of individual antibiotic exposure with depressive symptoms.

Figure S2 Nonlinear associations between exposure to different types of antibiotics and depressive symptoms.

Figure S3 Distribution of short-chain organic acids in the elderly.

Figure S4. Nonlinear association between serum short-chain organic acids and risk of depression in the elderly.

Figure S5. Intermediation of BHB.

Table S1. The physicochemical information of short-chain organic acids.

Metabolites	Formula	Molecular weight
AA	$C_2H_4O_2$	60.05
PA	$C_3H_6O_2$	74.08
Crotonic acid	$C_4H_6O_2$	86.09
iso-BA	$C_4H_8O_2$	88.11
BA	$C_4H_8O_2$	88.11
iso-VA	$C_5H_{10}O_2$	102.13
VA	$C_5H_{10}O_2$	102.13
iso-CA	$C_6H_{12}O_2$	116.16
CA	$C_6H_{12}O_2$	116.16
LA	$C_3H_6O_3$	90.08
BHB	$C_4H_8O_3$	104.10

AA, Acetic Acid; PA, Propionic Acid; BA, Butyric Acid; VA, Valeric Acid; CA, Caproic Acid; LA, Lactic Acid; BHB, β-hydroxybutyric Acid

Table S2. Detection frequency and urinary concentration (μg/g) of individual antibiotics.

Antibiotics	Usage	*n* [a]	% [b]	Percentiles [c]				Maximum [c]
				P_{50}	P_{75}	P_{95}	P_{99}	
**Macrolides**								
Azithromycin	HAs	179	18.2	<LOD	<LOD	0.24	5.65	981.49
Erythromycin	PHAs	84	8.5	<LOD	<LOD	1.40	16.14	4965.25
Roxithromycin	HAs	27	2.7	<LOD	<LOD	<LOD	0.29	1661.98
Clarithromycin	HAs	27	2.7	<LOD	<LOD	<LOD	0.24	4.26
**β-Lactams**								
Penicillin V	PHAs	176	17.9	<LOD	<LOD	2.36	9.73	163.63
Amoxicillin	PHAs	77	7.8	<LOD	<LOD	1.05	220.57	13520.02
Cefaclor	HAs	3	0.3	<LOD	<LOD	<LOD	<LOD	9.60
Cefotaxime	HAs	8	0.8	<LOD	<LOD	<LOD	<LOD	24.00
**Sulfonamides**								
Sulfaclozine	VAs	349	35.5	<LOD	4.00	22.08	54.52	680.56
Trimethoprime d	PVAs	200	20.3	<LOD	<LOD	1.65	13.32	29803.34
Sulfamonomethoxine e	VAs	64	6.5	<LOD	<LOD	0.68	4.77	68.61
Sulfachloropyridazine	VAs	44	4.5	<LOD	<LOD	<LOD	2.03	28.71
Sulfadiazine	PVAs	28	2.8	<LOD	<LOD	<LOD	1.68	120.19
Sulfamethoxazole	PVAs	26	2.6	<LOD	<LOD	<LOD	6.51	21964.17
Sulfamethazine	VAs	6	0.6	<LOD	<LOD	<LOD	<LOD	5.90
**Tetracyclines**								
Tetracycline	PVAs	190	19.3	<LOD	<LOD	1.96	17.16	1042.67
Oxytetracycline	PVAs	186	18.9	<LOD	<LOD	1.83	569.89	30168.85
Doxycycline	PVAs	181	18.4	<LOD	<LOD	1.57	10.54	80.34
Chlortetracycline	PVAs	77	7.8	<LOD	<LOD	0.97	8.89	906.36
**Fluoroquinolones**								
Ofloxacin	PVAs	231	23.5	<LOD	<LOD	3.94	42.85	113.95
Ciprofloxacin	PVAs	163	16.6	<LOD	<LOD	4.41	35.11	122.53
Norfloxacin	PVAs	117	11.9	<LOD	<LOD	4.63	252.35	62670.01
Enrofloxacin	VAs	103	10.5	<LOD	<LOD	0.67	7.47	22.43
Pefloxacin	PVAs	40	4.1	<LOD	<LOD	<LOD	5.12	342.50
Danofloxacin	VAs	40	4.1	<LOD	<LOD	<LOD	19.48	158397.90
Levofloxacin	HAs	33	3.4	<LOD	<LOD	<LOD	156.81	179826.37
Sarafloxacin	VAs	16	1.6	<LOD	<LOD	<LOD	0.29	214.82
Lomefloxacin	PVAs	14	1.4	<LOD	<LOD	<LOD	0.55	3656.44
Difloxacin	VAs	7	0.7	<LOD	<LOD	<LOD	<LOD	13.00
**Chloramphenicols**								
Florfenicol [e]	VAs	227	23.1	<LOD	<LOD	4.73	11.78	111.93
Chloramhenicol	HAs	27	2.7	<LOD	<LOD	<LOD	79.13	1811.49
Thiamphenicol	PVAs	2	0.2	<LOD	<LOD	<LOD	<LOD	203.85
**Lincosamides**								
Lincomycin	PVAs	36	3.7	<LOD	<LOD	<LOD	171.89	194642.29
**Cyadox**								
Cyadox	VAs	29	2.9	<LOD	<LOD	<LOD	12.23	79.30

[a] The number of subjects exposured to antibiotics;

[b] The detection frequency of antibiotics;

[c] Creatinine correction concentration (μg/g);

[d] Due to the similar antibacterial mechanisms, trimethoprim was included in the sulfonamides;

[e] The urinary levels of sulfamonomethoxine and florfenicol were separately considered to be the sum of their prototypes and metabolites (sulfamonomethoxine-N4-acetyl and florfenicol amine);

HAs, Human Antibiotics; VAs, Veterinary Antibiotics; PHAs, Antibiotics Preferred as HAs; PVAs, Antibiotics Preferred as VAs.

Table S3. Detection frequency and urinary concentration (μg/g) of various antibiotics.

Antibiotics	*n* [a]	% [b]	Percentiles [c]				Maximum [c]
			P_{50}	P_{75}	P_{95}	P_{99}	
**Chemical construction**							
Sulfonamides	548	55.7	0.36	5.16	28.05	132.70	51770.05
β-Lactams	253	25.7	<LOD	0.23	5.87	221.56	13520.02
Macrolides	280	28.5	<LOD	0.05	2.57	26.06	4966.18
Fluoroquinolones	491	49.9	<LOD	3.04	65.65	1234.86	179829.84
Tetracyclines	437	44.4	<LOD	1.03	7.44	608.34	30574.33
Chloramphenicols	246	25.0	<LOD	0.25	6.84	90.84	1811.49
**Usage**							
HAs	270	27.4	<LOD	0.04	22.71	690.37	179826.41
PHAs	305	31.0	<LOD	0.55	8.99	346.07	13520.02
VAs	618	62.8	1.98	7.12	31.05	98.32	158437.71
PVAs	716	72.8	1.29	5.44	90.69	15085.17	194958.32
**Antibacterial Spectrum**							
Broad-spectrum antibiotics	585	59.5	10.41	24.67	320.49	20605.24	179918.50
Narrow-spectrum antibiotics	238	24.2	<LOD	<LOD	5.09	221.58	13520.12
**Mode of action**							
Bactericidal antibiotics	362	36.8	<LOD	5.71	101.26	6951.52	179830.63
Bacteriostatic antibiotics	524	53.3	6.16	12.98	78.64	2809.97	51774.42

[a] The number of subjects exposured to antibiotics;
[b] The detection frequency of antibiotics;
[c] Creatinine correction concentration (μg/g);
HAs, Human Antibiotics; VAs, Veterinary Antibiotics; PHAs, Antibiotics Preferred as HAs; PVAs, Antibiotics Preferred as VAs.

Table S4. Associations between per lg10 increment of antibiotic concentrations and GDS-30 (*n* = 984).

**Antibiotics**	**Model 1** [a]		**Model 2** [b]	
	***β* (95% *CI*)**	***P*-value**	***β* (95% *CI*)**	***P*-value**
**Individual**				
Sulfaclozine	**0.456 (0.034, 0.878)**	**0.034**	**0.456 (0.034, 0.878)**	**0.041**
Trimethoprim	-0.181 (-0.779, 0.417)	0.553	-0.251 (-0.801, 0.299)	0.370
Azithromycin	0.073 (-0.750, 0.896)	0.862	0.345 (-0.412, 1.102)	0.372
Penicillin V	-0.394 (-1.228, 0.441)	0.355	-0.682 (-1.453, 0.089)	0.083
Ofloxacin	0.298 (-0.189, 0.785)	0.230	0.142 (-0.306, 0.591)	0.534
Enrofloxacin	0.069 (-0.706, 0.844)	0.861	0.014 (-0.700, 0.727)	0.970
Ciprofloxacin	0.469 (-0.058, 0.996)	0.081	0.451 (-0.033, 0.935)	0.068
Norfloxacin	**0.523 (0.062, 0.984)**	**0.026**	0.339 (-0.086, 0.763)	0.118
Oxytetracycline	**0.644 (0.017, 1.270)**	**0.044**	0.269 (-0.312, 0.850)	0.364
Tetracycline	0.505 (-0.287, 1.297)	0.211	0.228 (-0.500, 0.957)	0.538
Doxycycline	-0.270 (-1.158, 0.618)	0.551	0.082 (-0.734, 0.899)	0.843
Florfenicol	0.472 (-0.068, 1.011)	0.087	0.497 (-0.0005, 0.994)	0.050
**Category**				
Sulfonamides	**0.687 (0.081, 1.294)**	**0.026**	0.472 (-0.089, 1.032)	0.099
β-Lactams	0.221 (-0.559, 1.001)	0.578	-0.017 (-0.737, 0.703)	0.963
Macrolides	0.173 (-0.599, 0.944)	0.661	0.235 (-0.474, 0.944)	0.515
Fluoroquinolones	0.35 (-0.107, 0.806)	0.133	0.163 (-0.258, 0.583)	0.448
Tetracyclines	0.384 (-0.279, 1.047)	0.256	0.254 (-0.355, 0.863)	0.413
Chloramphenicols	0.869 (-0.429, 2.167)	0.189	0.895 (-0.301, 2.091)	0.141
**Usage**				
HAs	-0.083 (-0.782, 0.616)	0.817	-0.174 (-0.816, 0.469)	0.596
PHAs	0.13 (-0.466, 0.726)	0.669	0.033 (-0.516, 0.583)	0.905
VAs	**0.797 (0.075, 1.519)**	**0.031**	**0.666 (0.002, 1.329)**	**0.049**
PVAs	**0.717 (0.103, 1.331)**	**0.022**	0.201 (-0.370, 0.773)	0.489
**Antibacterial Spectrum**				
Broad-spectrum antibiotics	**0.636 (0.029, 1.243)**	**0.040**	0.223 (-0.339, 0.785)	0.437
Narrow-spectrum antibiotics	0.012 (-0.595, 0.619)	0.970	-0.171 (-0.732, 0.390)	0.549
**Mode of action**				
Bactericidal antibiotics	0.346 (-0.159, 0.851)	0.179	0.122 (-0.344, 0.588)	0.608
Bacteriostatic antibiotics	**0.732 (0.112, 1.352)**	**0.021**	0.397 (-0.176, 0.970)	0.174

[a] Model was analyzed by multiple linear regression;
Model 1 was adjusted for age, gender;
Model 2 was adjusted for age, gender, living alone, educational level, marital status, physical activity, drinking, cognitive impairment, and dietary structure;
Bolding indicates *P* value < 0.05;
GDS-30, Geriatric Depression Scale-30; HAs, Human Antibiotics; VAs, Veterinary Antibiotics; PHAs, Antibiotics Preferred as HAs; PVAs, Antibiotics Preferred as VAs; β, Correlation Coefficient; CI：Confidence Interval.

Table S5. Associations of creatinine-adjusted urinary individual antibiotics with depression in the elderly by multinomial logistic regression (less than LODs was used as the control) (*n* = 984).

Antibiotics	Model 1 [a]			Model 2 [a]		
	Ref	Low	High	Ref	Low	High
**Individual**						
Sulfaclozine	Ref	0.950 (0.646, 1.399)	**1.549 (1.079, 2.222)**	Ref	0.900 (0.592, 1.366)	**1.557 (1.059, 2.291)**
Trimethoprim	Ref	0.838 (0.515, 1.366)	0.829 (0.513, 1.342)	Ref	0.873 (0.518, 1.471)	0.828 (0.495, 1.385)
Azithromycin	Ref	0.652 (0.379, 1.119)	1.331 (0.835, 2.119)	Ref	0.832 (0.468, 1.479)	**1.701 (1.022, 2.831)**
Penicillin V	Ref	0.623 (0.362, 1.071)	0.933 (0.569, 1.532)	Ref	0.862 (0.481, 1.546)	0.700 (0.410, 1.197)
Ofloxacin	Ref	1.327 (0.870, 2.026)	1.079 (0.698, 1.670)	Ref	1.113 (0.710, 1.745)	1.022 (0.641, 1.629)
Enrofloxacin	Ref	0.853 (0.437, 1.666)	1.110 (0.601, 2.049)	Ref	0.870 (0.424, 1.785)	1.041 (0.543, 1.997)
Ciprofloxacin	Ref	1.229 (0.746, 2.022)	1.348 (0.827, 2.198)	Ref	1.219 (0.713, 2.086)	1.370 (0.810, 2.317)
Norfloxacin	Ref	1.368 (0.772, 2.425)	1.548 (0.887, 2.704)	Ref	1.408 (0.763, 2.597)	1.348 (0.740, 2.457)
Oxytetracycline	Ref	0.953 (0.583, 1.558)	**1.788 (1.140, 2.802)**	Ref	0.981 (0.583, 1.649)	1.457 (0.894, 2.376)
Tetracycline	Ref	1.178 (0.735, 1.889)	1.366 (0.859, 2.172)	Ref	1.071 (0.645, 1.778)	1.423 (0.861, 2.353)
Doxycycline	Ref	0.816 (0.492, 1.354)	0.969 (0.594, 1.582)	Ref	0.906 (0.526, 1.559)	1.077 (0.634, 1.829)
Florfenicol	Ref	**1.561 (1.025, 2.377)**	1.096 (0.707, 1.700)	Ref	1.536 (0.978, 2.413)	1.158 (0.722, 1.859)

[a] Model was analyzed by binary logistic regression;
Model 1 was adjusted for age, gender;
Model 2 was adjusted for age, gender, living alone, educational level, marital status, physical activity, drinking, cognitive impairment, and dietary structure;
Bolding indicates *P* value < 0.05;
HAs, Human Antibiotics; VAs, Veterinary Antibiotics; PHAs, Antibiotics Preferred as HAs; PVAs, Antibiotics Preferred as VAs; OR, Odds Ratio; CI, Confidence Interval; BMI, Body Mass Index; ADL, Activities of Daily Living; Ref, Reference.

Table S6. Associations of creatinine-adjusted urinary various antibiotics with depression in the elderly by multinomial logistic regression (less than LODs was used as the control) (n = 984).

Antibiotics	Model 1 [a]			Model 2 [a]		
	Ref	Low	High	Ref	Low	High
**Chemical structure**						
Sulfonamides	Ref	0.833 (0.586, 1.185)	1.263 (0.907, 1.759)	Ref	0.872 (0.599, 1.269)	1.184 (0.831, 1.686)
β-Lactams	Ref	0.662 (0.419, 1.045)	1.066 (0.704, 1.615)	Ref	0.799 (0.489, 1.304)	0.879 (0.563, 1.373)
Macrolides	Ref	0.801 (0.525, 1.220)	0.904 (0.600, 1.361)	Ref	1.059 (0.671, 1.672)	0.986 (0.636, 1.530)
Fluoroquinolones	Ref	1.270 (0.902, 1.789)	1.318 (0.938, 1.852)	Ref	1.158 (0.803, 1.670)	1.214 (0.843, 1.749)
Tetracyclines	Ref	1.132 (0.796, 1.610)	1.333 (0.943, 1.884)	Ref	1.189 (0.818, 1.729)	**1.468 (1.008, 2.138)**
Chloramphenicols	Ref	**1.603 (1.067, 2.408)**	1.012 (0.658, 1.555)	Ref	1.496 (0.967, 2.313)	1.048 (0.661, 1.660)
**Usage**						
HAs	Ref	0.726 (0.470, 1.121)	0.795 (0.520, 1.214)	Ref	0.942 (0.590, 1.505)	0.822 (0.525, 1.289)
PHAs	Ref	0.730 (0.482, 1.105)	0.986 (0.667, 1.456)	Ref	0.898 (0.575, 1.402)	0.862 (0.566, 1.311)
VAs	Ref	1.221 (0.862, 1.728)	**1.459 (1.040, 2.048)**	Ref	1.192 (0.823, 1.726)	**1.477 (1.028, 2.123)**
PVAs	Ref	1.113 (0.773, 1.604)	1.400 (0.978, 2.003)	Ref	1.214 (0.822, 1.791)	1.334 (0.911, 1.956)
**Antibacterial Spectrum**						
Broad-spectrum antibiotics	Ref	**1.470 (1.042, 2.074)**	**1.486 (1.057, 2.089)**	Ref	**1.608 (1.112, 2.326)**	1.435 (0.998, 2.065)
Narrow-spectrum antibiotics	Ref	0.644 (0.402, 1.031)	0.981 (0.638, 1.508)	Ref	0.754 (0.455, 1.251)	0.782 (0.492, 1.242)
**Mode of action**						
Bactericidal antibiotics	Ref	1.338 (0.932, 1.921)	1.052 (0.725, 1.527)	Ref	1.239 (0.841, 1.826)	0.936 (0.628, 1.395)
Bacteriostatic antibiotics	Ref	0.941 (0.642, 1.377)	1.369 (0.981, 1.912)	Ref	0.999 (0.664, 1.501)	1.378 (0.965, 1.967)

[a] Model was analyzed by binary logistic regression;
Model 1 was adjusted for age, gender;
Model 2 was adjusted for age, gender, living alone, educational level, marital status, physical activity, drinking, cognitive impairment, and dietary structure;
Bolding indicates *P* value < 0.05;
HAs, Human Antibiotics; VAs, Veterinary Antibiotics; PHAs, Antibiotics Preferred as HAs; PVAs, Antibiotics Preferred as VAs; OR, Odds Ratio; CI, Confidence Interval; BMI, Body Mass Index; ADL, Activities of Daily Living; Ref, Reference.

Table S7. Distribution of short chain organic acids in serum.

Metabolites	%>LOD	Median	Geometric mean	Range
AA (nmol/L)	100	30876.07	26147.13	84.65 ~ 322813.08
PA (nmol/L)	100	241.79	245.21	46.15 ~ 1932.66
Crotonic acid (nmol/L)	67.9	11.42	12.14	5.83 ~ 61.51
iso-BA (nmol/L)	100	348.28	325.31	1.40 ~ 4413.48
BA (nmol/L)	100	59.41	62.85	17.05 ~ 482.17
iso-VA (nmol/L)	100	529.76	473.90	0.11 ~ 6817.20
VA (nmol/L)	100	9.25	8.48	0.03 ~ 145.30
iso-CA (nmol/L)	100	0.89	0.88	0.07 ~ 4.16
CA (nmol/L)	100	752.61	706.58	25.34 ~ 7261.49
LA (mmol/L)	100	3.66	3.68	1.03 ~ 14.00
BHB (μmol/L)	100	93.05	94.21	21.41 ~ 831.24

AA, Acetic Acid; PA, Propionic Acid; BA, Butyric Acid; VA, Valeric Acid; CA, Caproic Acid; LA, Lactic Acid; BHB, β-hydroxybutyric Acid; LOD, Limit of Detection.

Table S8. Association between urinary single antibiotic levels and serum short chain organic acids in the elderly.

Metabolites	Model	*β* (95% *CI*) [a]					
		Sulfaclozine	Trimethoprim	Azithromycin	Penicillin V	Ofloxacin	Enrofloxacin
AA	Model 1	**0.042 (0.011, 0.073)**	0.013 (-0.031, 0.057)	-0.052 (-0.112, 0.008)	-0.039 (-0.100, 0.022)	0.007 (-0.029, 0.043)	-0.001 (-0.058, 0.056)
	Model 2	**0.037 (0.006, 0.068)**	0.017 (-0.027, 0.061)	-0.050 (-0.110, 0.010)	-0.034 (-0.096, 0.027)	0.010 (-0.025, 0.046)	0.002 (-0.055, 0.059)
PA	Model 1	-0.002 (-0.016, 0.011)	-0.003 (-0.022, 0.016)	**-0.029 (-0.056, -0.003)**	-0.003 (-0.030, 0.024)	-0.012 (-0.028, 0.003)	**0.029 (0.004, 0.054)**
	Model 2	-0.003 (-0.017, 0.010)	-0.004 (-0.023, 0.015)	**-0.030 (-0.056, -0.003)**	-0.008 (-0.035, 0.019)	-0.013 (-0.029, 0.002)	**0.027 (0.002, 0.051)**
Crotonic acid	Model 1	-0.015 (-0.036, 0.007)	-0.020 (-0.050, 0.011)	-0.0001 (-0.042, 0.042)	0.017 (-0.025, 0.060)	-0.003 (-0.027, 0.022)	-0.013 (-0.052, 0.027)
	Model 2	-0.016 (-0.037, 0.006)	-0.019 (-0.050, 0.011)	0.002 (-0.040, 0.044)	0.018 (-0.025, 0.061)	-0.003 (-0.028, 0.022)	-0.013 (-0.053, 0.027)
iso-BA	Model 1	-0.023 (-0.053, 0.007)	-0.011 (-0.053, 0.031)	-0.024 (-0.082, 0.033)	-0.053 (-0.111, 0.005)	**-0.042 (-0.076, -0.008)**	0.009 (-0.045, 0.063)
	Model 2	-0.025 (-0.054, 0.005)	-0.015 (-0.057, 0.027)	-0.023 (-0.080, 0.035)	-0.058 (-0.117, 0.001)	**-0.042 (-0.076, -0.008)**	0.010 (-0.045, 0.064)
BA	Model 1	-0.012 (-0.025, 0.001)	-0.016 (-0.034, 0.002)	**-0.026 (-0.051, -0.0002)**	0.006 (-0.02, 0.032)	-0.007 (-0.022, 0.008)	0.005 (-0.019, 0.029)
	Model 2	-0.013 (-0.026, 0.00002)	-0.017 (-0.036, 0.001)	**-0.025 (-0.050, -0.0002)**	0.002 (-0.024, 0.027)	-0.007 (-0.022, 0.008)	0.004 (-0.020, 0.028)
iso-VA	Model 1	-0.029 (-0.061, 0.004)	-0.021 (-0.067, 0.025)	-0.026 (-0.089, 0.038)	0.007 (-0.057, 0.071)	-0.031 (-0.068, 0.006)	0.025 (-0.034, 0.085)
	Model 2	-0.032 (-0.065, 0.001)	-0.019 (-0.065, 0.028)	-0.023 (-0.086, 0.040)	0.008 (-0.056, 0.073)	-0.031 (-0.069, 0.006)	0.027 (-0.032, 0.087)
VA	Model 1	-0.014 (-0.044, 0.017)	-0.038 (-0.081, 0.006)	-0.048 (-0.107, 0.012)	**-0.071 (-0.132, -0.011)**	0.0002 (-0.035, 0.036)	-0.014 (-0.070, 0.043)
	Model 2	-0.022 (-0.052, 0.009)	-0.036 (-0.079, 0.007)	-0.045 (-0.105, 0.014)	**-0.071 (-0.131, -0.011)**	-0.002 (-0.037, 0.033)	-0.017 (-0.073, 0.039)
iso-CA	Model 1	-0.016 (-0.033, 0.001)	0.017 (-0.007, 0.040)	-0.016 (-0.049, 0.016)	-0.001 (-0.034, 0.031)	**-0.025 (-0.044, -0.006)**	0.003 (-0.028, 0.033)
	Model 2	-0.014 (-0.031, 0.002)	0.017 (-0.006, 0.040)	-0.023 (-0.054, 0.009)	0.004 (-0.028, 0.037)	**-0.021 (-0.039, -0.002)**	0.005 (-0.025, 0.035)
CA	Model 1	-0.00006 (-0.021, 0.020)	-0.018 (-0.047, 0.011)	**-0.061 (-0.101, -0.021)**	-0.006 (-0.046, 0.034)	-0.008 (-0.031, 0.016)	-0.020 (-0.058, 0.017)
	Model 2	-0.006 (-0.026, 0.014)	-0.017 (-0.046, 0.011)	**-0.055 (-0.094, -0.017)**	-0.013 (-0.053, 0.027)	-0.011 (-0.034, 0.012)	-0.024 (-0.061, 0.013)
LA	Model 1	**0.020 (0.007, 0.033)**	0.013 (-0.005, 0.031)	-0.002 (-0.028, 0.023)	-0.019 (-0.045, 0.007)	0.010 (-0.005, 0.025)	0.015 (-0.009, 0.039)
	Model 2	**0.022 (0.009, 0.035)**	0.013 (-0.005, 0.032)	-0.004 (-0.029, 0.022)	-0.020 (-0.046, 0.006)	0.012 (-0.003, 0.027)	0.015 (-0.008, 0.039)
BHB	Model 1	**0.020 (0.005, 0.035)**	0.0003 (-0.021, 0.022)	0.016 (-0.013, 0.045)	-0.019 (-0.048, 0.011)	0.009 (-0.008, 0.027)	0.011 (-0.017, 0.038)
	Model 2	**0.021 (0.006, 0.036)**	0.0003 (-0.021, 0.022)	0.013 (-0.016, 0.043)	-0.016 (-0.046, 0.014)	0.012 (-0.006, 0.029)	0.010 (-0.017, 0.038)

[a] Model was analyzed by multiple linear regression;
Model 1 was adjusted for age, gender;
Model 2 was adjusted for age, gender, living alone, educational level, marital status, physical activity, drinking, cognitive impairment, and dietary structure；
Bolding indicates *P* value < 0.05;
AA, Acetic Acid; PA, Propionic Acid; BA, Butyric Acid; VA, Valeric Acid; CA, Caproic Acid; LA, Lactic Acid; BHB, β-hydroxybutyric Acid; β, Correlation Coefficient; CI, Confidence Interval.

Continued.

Metabolites	Model	*β* (95% *CI*) [a]					
		**Ciprofloxacin**	**Norfloxacin**	**Oxytetracycline**	**Tetracycline**	**Doxycycline**	**Florfenicol**
AA	Model 1	0.006 (-0.033, 0.045)	-0.003 (-0.037, 0.031)	-0.049 (-0.095, -0.003)	-0.051 (-0.109, 0.007)	**0.132 (0.068, 0.197)**	-0.014 (-0.054, 0.026)
	Model 2	0.009 (-0.030, 0.047)	-0.003 (-0.036, 0.031)	-0.043 (-0.089, 0.003)	-0.047 (-0.105, 0.011)	**0.131 (0.067, 0.196)**	-0.008 (-0.047, 0.032)
PA	Model 1	**0.022 (0.005, 0.039)**	-0.008 (-0.023, 0.007)	-0.009 (-0.030, 0.011)	-0.003 (-0.022, 0.016)	0.022 (-0.006, 0.051)	-0.001 (-0.018, 0.017)
	Model 2	**0.022 (0.005, 0.039)**	-0.009 (-0.024, 0.006)	-0.011 (-0.031, 0.009)	-0.020 (-0.046, 0.005)	0.026 (-0.003, 0.054)	-0.0003 (-0.018, 0.017)
crotonic acid	Model 1	0.006 (-0.021, 0.033)	0.022 (-0.002, 0.046)	-0.019 (-0.051, 0.013)	**-0.057 (-0.098, -0.017)**	0.041 (-0.004, 0.087)	0.007 (-0.021, 0.034)
	Model 2	0.007 (-0.020, 0.034)	0.022 (-0.002, 0.045)	-0.019 (-0.052, 0.013)	**-0.058 (-0.098, -0.017)**	0.043 (-0.002, 0.088)	0.009 (-0.019, 0.037)
iso-BA	Model 1	0.014 (-0.023, 0.051)	-0.015 (-0.047, 0.017)	**-0.053 (-0.097, -0.009)**	**-0.059 (-0.114, -0.004)**	0.056 (-0.006, 0.118)	0.007 (-0.031, 0.044)
	Model 2	0.014 (-0.023, 0.051)	-0.017 (-0.049, 0.016)	**-0.054 (-0.098, -0.01)**	**-0.061 (-0.117, -0.006)**	0.059 (-0.004, 0.121)	0.007 (-0.031, 0.045)
BA	Model 1	0.010 (-0.006, 0.026)	0.004 (-0.010, 0.018)	-0.016 (-0.035, 0.003)	-0.019 (-0.043, 0.006)	-0.010 (-0.037, 0.017)	**0.018 (0.001, 0.035)**
	Model 2	0.010 (-0.006, 0.026)	0.003 (-0.012, 0.017)	-0.017 (-0.037, 0.002)	-0.02 (-0.045, 0.004)	-0.007 (-0.035, 0.020)	**0.018 (0.002, 0.035)**
iso-VA	Model 1	0.027 (-0.013, 0.068)	-0.010 (-0.046, 0.025)	0.003 (-0.045, 0.051)	0.018 (-0.043, 0.078)	0.066 (-0.002, 0.134)	-0.018 (-0.059, 0.024)
	Model 2	0.028 (-0.013, 0.068)	-0.011 (-0.046, 0.025)	0.001 (-0.048, 0.050)	0.018 (-0.043, 0.079)	0.068 (-0.001, 0.136)	-0.016 (-0.057, 0.026)
VA	Model 1	0.014 (-0.025, 0.052)	-0.024 (-0.058, 0.009)	-0.014 (-0.060, 0.032)	-0.036 (-0.094, 0.021)	0.009 (-0.056, 0.073)	0.013 (-0.026, 0.052)
	Model 2	0.013 (-0.025, 0.051)	-0.027 (-0.060, 0.006)	-0.019 (-0.064, 0.026)	-0.038 (-0.094, 0.019)	0.013 (-0.050, 0.077)	0.021 (-0.018, 0.060)
iso-CA	Model 1	-0.008 (-0.029, 0.012)	-0.010 (-0.029, 0.008)	-0.025 (-0.050, -0.0005)	**-0.067 (-0.098, -0.036)**	0.016 (-0.019, 0.051)	0.014 (-0.007, 0.035)
	Model 2	-0.008 (-0.029, 0.012)	-0.007 (-0.024, 0.011)	-0.016 (-0.040, 0.008)	**-0.061 (-0.091, -0.031)**	0.007 (-0.027, 0.041)	0.013 (-0.008, 0.033)
CA	Model 1	0.004 (-0.022, 0.029)	0.021 (-0.002, 0.043)	0.019 (-0.011, 0.050)	-0.023 (-0.061, 0.016)	0.028 (-0.015, 0.071)	-0.016 (-0.043, 0.010)
	Model 2	0.004 (-0.021, 0.029)	0.016 (-0.006, 0.038)	0.012 (-0.018, 0.041)	-0.028 (-0.065, 0.010)	0.038 (-0.004, 0.080)	-0.012 (-0.037, 0.014)
LA	Model 1	0.002 (-0.014, 0.018)	-0.008 (-0.022, 0.006)	0.01 (-0.010, 0.029)	**0.033 (0.009, 0.058)**	**0.037 (0.009, 0.064)**	0.013 (-0.004, 0.030)
	Model 2	0.003 (-0.013, 0.019)	-0.006 (-0.020, 0.008)	0.013 (-0.006, 0.033)	**0.035 (0.011, 0.060)**	**0.035 (0.008, 0.062)**	0.012 (-0.005, 0.029)
BHB	Model 1	-0.004 (-0.022, 0.015)	0.001 (-0.016, 0.017)	-0.009 (-0.031, 0.014)	-0.005 (-0.033, 0.023)	**0.035 (0.004, 0.067)**	-0.014 (-0.034, 0.005)
	Model 2	-0.003 (-0.022, 0.016)	0.002 (-0.015, 0.018)	-0.003 (-0.025, 0.020)	-0.002 (-0.030, 0.026)	**0.032 (0.001, 0.064)**	-0.013 (-0.033, 0.006)

[a] Model was analyzed by multiple linear regression;

Model 1 was adjusted for age, gender;

Model 2 was adjusted for age, gender, living alone, educational level, marital status, physical activity, drinking, cognitive impairment, and dietary structure；

Bolding indicates *P* value < 0.05;

AA, Acetic Acid; PA, Propionic Acid; BA, Butyric Acid; VA, Valeric Acid; CA, Caproic Acid; LA, Lactic Acid; BHB, β-hydroxybutyric Acid; β, Correlation Coefficient; CI, Confidence Interval.

Table S9. Association between urinary levels of various antibiotics (based on chemical structure) and serum short-chain organic acids in the elderly.

Metabolites	Model	***β (95% CI)*** [a]					
		**Sulfonamides**	**β-Lactams**	**Macrolides**	**Fluoroquinolones**	**Tetracyclines**	**Chloramphenicols**
AA	Model 1	**0.056 (0.012, 0.101)**	0.017 (-0.040, 0.075)	**-0.069 (-0.126, -0.013)**	0.017 (-0.016, 0.051)	-0.014 (-0.062, 0.035)	-0.021 (-0.116, 0.075)
	Model 2	**0.053 (0.008, 0.097)**	0.021 (-0.036, 0.078)	**-0.067 (-0.124, -0.011)**	0.020 (-0.014, 0.053)	-0.009 (-0.058, 0.039)	-0.012 (-0.107, 0.083)
PA	Model 1	0.001 (-0.019, 0.020)	0.014 (-0.011, 0.039)	-0.006 (-0.031, 0.019)	0.002 (-0.012, 0.017)	-0.002 (-0.023, 0.02)	-0.016 (-0.058, 0.025)
	Model 2	-0.002 (-0.021, 0.018)	0.014 (-0.011, 0.039)	-0.007 (-0.031, 0.018)	0.001 (-0.014, 0.016)	-0.002 (-0.023, 0.019)	-0.016 (-0.058, 0.025)
crotonic acid	Model 1	-0.016 (-0.047, 0.015)	-0.003 (-0.043, 0.037)	-0.029 (-0.068, 0.011)	0.018 (-0.005, 0.042)	-0.012 (-0.046, 0.022)	-0.012 (-0.079, 0.054)
	Model 2	-0.017 (-0.048, 0.014)	-0.002 (-0.042, 0.038)	-0.027 (-0.067, 0.012)	0.018 (-0.006, 0.041)	-0.011 (-0.045, 0.022)	-0.008 (-0.074, 0.059)
iso-BA	Model 1	-0.022 (-0.065, 0.020)	-0.007 (-0.061, 0.048)	0.035 (-0.019, 0.089)	-0.027 (-0.059, 0.005)	-0.038 (-0.085, 0.008)	0.031 (-0.060, 0.122)
	Model 2	-0.026 (-0.069, 0.017)	-0.006 (-0.061, 0.049)	0.035 (-0.019, 0.089)	-0.028 (-0.060, 0.004)	-0.038 (-0.084, 0.009)	0.032 (-0.060, 0.123)
BA	Model 1	-0.016 (-0.035, 0.002)	-0.001 (-0.025, 0.023)	-0.002 (-0.026, 0.021)	-0.003 (-0.017, 0.011)	-0.007 (-0.028, 0.013)	0.003 (-0.037, 0.043)
	Model 2	**-0.019 (-0.038, -0.00003)**	-0.001 (-0.025, 0.023)	-0.003 (-0.027, 0.021)	-0.004 (-0.018, 0.010)	-0.008 (-0.028, 0.013)	0.002 (-0.039, 0.042)
iso-VA	Model 1	-0.038 (-0.084, 0.009)	0.017 (-0.043, 0.077)	0.017 (-0.042, 0.076)	-0.026 (-0.061, 0.009)	0.030 (-0.021, 0.081)	0.007 (-0.093, 0.106)
	Model 2	-0.041 (-0.088, 0.006)	0.013 (-0.047, 0.074)	0.019 (-0.041, 0.078)	-0.025 (-0.061, 0.010)	0.030 (-0.021, 0.081)	0.010 (-0.090, 0.110)
VA	Model 1	-0.026 (-0.070, 0.018)	0.005 (-0.052, 0.061)	0.019 (-0.037, 0.075)	-0.005 (-0.039, 0.028)	-0.019 (-0.067, 0.029)	0.040 (-0.054, 0.134)
	Model 2	-0.038 (-0.082, 0.006)	0.003 (-0.053, 0.059)	0.020 (-0.035, 0.075)	-0.007 (-0.040, 0.026)	-0.018 (-0.066, 0.029)	0.055 (-0.039, 0.148)
iso-CA	Model 1	-0.014 (-0.037, 0.010)	0.006 (-0.025, 0.036)	-0.010 (-0.040, 0.020)	-0.017 (-0.035, 0.001)	**-0.031 (-0.057, -0.005)**	0.007 (-0.044, 0.058)
	Model 2	-0.008 (-0.032, 0.015)	0.012 (-0.018, 0.042)	-0.013 (-0.042, 0.017)	-0.013 (-0.030, 0.005)	**-0.029 (-0.054, -0.004)**	0.003 (-0.047, 0.052)
CA	Model 1	0.009 (-0.020, 0.039)	0.002 (-0.036, 0.040)	-0.016 (-0.053, 0.022)	0.010 (-0.012, 0.032)	0.003 (-0.030, 0.035)	-0.015 (-0.077, 0.048)
	Model 2	-0.001 (-0.029, 0.028)	-0.003 (-0.040, 0.034)	-0.014 (-0.050, 0.022)	0.006 (-0.016, 0.027)	0.001 (-0.030, 0.033)	-0.007 (-0.069, 0.054)
LA	Model 1	**0.026 (0.007, 0.045)**	0.004 (-0.020, 0.028)	-0.005 (-0.029, 0.019)	0.002 (-0.013, 0.016)	**0.025 (0.004, 0.045)**	0.006 (-0.034, 0.047)
	Model 2	**0.029 (0.011, 0.048)**	0.006 (-0.018, 0.030)	-0.005 (-0.029, 0.018)	0.003 (-0.011, 0.017)	**0.026 (0.005, 0.046)**	0.004 (-0.036, 0.044)
BHB	Model 1	**0.023 (0.002, 0.045)**	0.002 (-0.026, 0.029)	-0.010 (-0.037, 0.018)	0.011 (-0.005, 0.028)	0.001 (-0.023, 0.025)	-0.037 (-0.083, 0.009)
	Model 2	**0.025 (0.004, 0.047)**	0.008 (-0.020, 0.036)	-0.011 (-0.039, 0.016)	0.013 (-0.004, 0.029)	0.003 (-0.021, 0.026)	-0.036 (-0.083, 0.010)

[a] Model was analyzed by multiple linear regression;
Model 1 was adjusted for age, gender;
Model 2 was adjusted for age, gender, living alone, educational level, marital status, physical activity, drinking, cognitive impairment, and dietary structure;
Bolding indicates *P* value < 0.05;
AA, Acetic Acid; PA, Propionic Acid; BA, Butyric Acid; VA, Valeric Acid; CA, Caproic Acid; LA, Lactic Acid; BHB, β-hydroxybutyric Acid; β, Correlation Coefficient; CI, Confidence Interval.

Table S10. Association of use-based levels of various antibiotics in urine with serum short-chain organic acids in the elderly.

Metabolites	Model	*β (95% CI)* [c]			
		**HAs**	**PHAs**	**VAs**	**PVAs**
AA	Model 1	-0.010 (-0.062, 0.041)	-0.019 (-0.063, 0.024)	0.049 (-0.004, 0.102)	-0.017 (-0.063, 0.028)
	Model 2	-0.007 (-0.058, 0.044)	-0.016 (-0.060, 0.027)	0.046 (-0.007, 0.099)	-0.010 (-0.055, 0.036)
PA	Model 1	-0.009 (-0.031, 0.014)	0.012 (-0.007, 0.031)	0.009 (-0.014, 0.033)	-0.004 (-0.024, 0.016)
	Model 2	-0.010 (-0.032, 0.013)	0.011 (-0.008, 0.030)	0.008 (-0.016, 0.031)	-0.006 (-0.026, 0.014)
crotonic acid	Model 1	-0.001 (-0.036, 0.035)	-0.009 (-0.040, 0.021)	-0.001 (-0.038, 0.036)	0.004 (-0.028, 0.035)
	Model 2	0.001 (-0.035, 0.037)	-0.001 (-0.038, 0.036)	-0.003 (-0.040, 0.034)	0.003 (-0.029, 0.035)
iso-BA	Model 1	-0.001 (-0.050, 0.048)	0.001 (-0.041, 0.043)	-0.005 (-0.056, 0.046)	**-0.051 (-0.094, -0.007)**
	Model 2	-0.001 (-0.050, 0.048)	0.0002 (-0.042, 0.042)	-0.007 (-0.058, 0.044)	**-0.053 (-0.096, -0.009)**
BA	Model 1	-0.013 (-0.034, 0.009)	0.004 (-0.015, 0.022)	-0.004 (-0.026, 0.018)	-0.014 (-0.033, 0.005)
	Model 2	-0.014 (-0.035, 0.008)	0.003 (-0.016, 0.021)	-0.006 (-0.028, 0.017)	-0.017 (-0.036, 0.002)
iso-VA	Model 1	-0.035 (-0.089, 0.019)	0.016 (-0.03, 0.062)	-0.023 (-0.079, 0.032)	-0.0005 (-0.048, 0.047)
	Model 2	-0.035 (-0.089, 0.019)	0.015 (-0.031, 0.061)	-0.024 (-0.080, 0.031)	0.0002 (-0.048, 0.048)
VA	Model 1	-0.016 (-0.067, 0.035)	0.017 (-0.027, 0.06)	-0.002 (-0.055, 0.05)	-0.013 (-0.057, 0.032)
	Model 2	-0.015 (-0.066, 0.035)	0.019 (-0.024, 0.061)	-0.007 (-0.059, 0.045)	-0.016 (-0.060, 0.029)
iso-CA	Model 1	-0.006 (-0.034, 0.021)	0.008 (-0.015, 0.032)	-0.020 (-0.048, 0.009)	**-0.040 (-0.064, -0.016)**
	Model 2	-0.005 (-0.032, 0.022)	0.010 (-0.013, 0.033)	-0.016 (-0.044, 0.011)	**-0.030 (-0.054, -0.006)**
CA	Model 1	0.008 (-0.026, 0.042)	0.001 (-0.028, 0.029)	-0.005 (-0.041, 0.03)	0.017 (-0.013, 0.047)
	Model 2	0.007 (-0.026, 0.040)	-0.0003 (-0.028, 0.028)	-0.011 (-0.045, 0.023)	0.007 (-0.022, 0.037)
LA	Model 1	0.004 (-0.017, 0.026)	-0.002 (-0.021, 0.016)	0.020 (-0.003, 0.042)	0.015 (-0.004, 0.034)
	Model 2	0.005 (-0.016, 0.027)	-0.002 (-0.021, 0.016)	0.021 (-0.001, 0.043)	0.019 (0.0004, 0.039)
BHB	Model 1	0.005 (-0.020, 0.030)	-0.014 (-0.036, 0.007)	**0.029 (0.003, 0.054)**	-0.008 (-0.030, 0.014)
	Model 2	0.006 (-0.019, 0.031)	-0.012 (-0.033, 0.010)	**0.029 (0.003, 0.055)**	-0.004 (-0.026, 0.018)

[a] Model was analyzed by multiple linear regression;
Model 1 was adjusted for age, gender;
Model 2 was adjusted for age, gender, living alone, educational level, marital status, physical activity, drinking, cognitive impairment, and dietary structure;
Bolding indicates *P* value < 0.05;
HAs, Human Antibiotics; VAs, Veterinary Antibiotics; PHAs, Antibiotics Preferred as HAs; PVAs, Antibiotics Preferred as VAs; AA, Acetic Acid; PA, Propionic Acid; BA, Butyric Acid; VA, Valeric Acid; CA, Caproic Acid; LA, Lactic Acid; BHB, β-hydroxybutyric Acid; β, Correlation Coefficient; CI, Confidence Interval.

Table S11. Association between urinary antibiotic levels (based on antimicrobial profiles and bactericidal mechanisms) and serum short-chain organic acids in the elderly.

Metabolites	Model	*β (95% CI)* [c]			
		**Bactericidal antibiotics**	**Bacteriostatic antibiotics**	**Broad-spectrum antibiotics**	**Narrow-spectrum antibiotics**
AA	Model 1	0.024 (-0.013, 0.061)	0.019 (-0.027, 0.065)	0.003 (-0.042, 0.047)	0.004 (-0.040, 0.049)
	Model 2	0.028 (-0.009, 0.065)	0.021 (-0.024, 0.067)	0.007 (-0.037, 0.052)	0.008 (-0.037, 0.053)
PA	Model 1	0.010 (-0.006, 0.026)	-0.004 (-0.024, 0.016)	-0.007 (-0.026, 0.013)	0.011 (-0.008, 0.031)
	Model 2	0.009 (-0.007, 0.025)	-0.007 (-0.027, 0.013)	-0.010 (-0.029, 0.010)	0.011 (-0.009, 0.030)
crotonic acid	Model 1	0.013 (-0.013, 0.039)	0.004 (-0.028, 0.036)	0.001 (-0.030, 0.032)	0.001 (-0.030, 0.032)
	Model 2	0.013 (-0.013, 0.039)	0.003 (-0.029, 0.035)	-0.0002 (-0.031, 0.031)	0.002 (-0.029, 0.033)
iso-BA	Model 1	-0.019 (-0.055, 0.016)	-0.021 (-0.064, 0.023)	**-0.044 (-0.086, -0.001)**	-0.011 (-0.054, 0.031)
	Model 2	-0.019 (-0.055, 0.016)	-0.024 (-0.068, 0.020)	**-0.047 (-0.090, -0.004)**	-0.012 (-0.055, 0.031)
BA	Model 1	-0.002 (-0.017, 0.014)	-0.015 (-0.034, 0.004)	-0.018 (-0.037, 0.001)	0.003 (-0.016, 0.021)
	Model 2	-0.002 (-0.018, 0.013)	-0.019 (-0.038, 0.001)	**-0.021 (-0.040, -0.002)**	0.001 (-0.017, 0.020)
iso-VA	Model 1	-0.019 (-0.058, 0.020)	**-0.049 (-0.096, -0.001)**	-0.038 (-0.085, 0.008)	0.011 (-0.036, 0.057)
	Model 2	-0.020 (-0.059, 0.019)	**-0.048 (-0.096, -0.0004)**	-0.038 (-0.085, 0.009)	0.009 (-0.038, 0.056)
VA	Model 1	0.002 (-0.035, 0.039)	-0.010 (-0.055, 0.035)	-0.023 (-0.067, 0.021)	-0.006 (-0.050, 0.038)
	Model 2	-0.00004 (-0.036, 0.036)	-0.014 (-0.059, 0.030)	-0.027 (-0.070, 0.017)	-0.005 (-0.049, 0.039)
iso-CA	Model 1	-0.010 (-0.030, 0.010)	-0.016 (-0.041, 0.008)	-0.024 (-0.048, 0.00007)	-0.024 (-0.048, 0.00007)
	Model 2	-0.004 (-0.024, 0.015)	-0.010 (-0.033, 0.014)	-0.015 (-0.039, 0.008)	0.010 (-0.013, 0.034)
CA	Model 1	0.015 (-0.009, 0.040)	0.013 (-0.018, 0.043)	0.006 (-0.024, 0.035)	0.004 (-0.025, 0.033)
	Model 2	0.011 (-0.013, 0.035)	0.004 (-0.026, 0.033)	-0.003 (-0.032, 0.025)	0.001 (-0.028, 0.030)
LA	Model 1	0.004 (-0.011, 0.020)	0.008 (-0.011, 0.027)	0.009 (-0.009, 0.028)	-0.001 (-0.02, 0.018)
	Model 2	0.006 (-0.010, 0.021)	0.010 (-0.009, 0.030)	0.012 (-0.006, 0.031)	-0.0004 (-0.019, 0.018)
BHB	Model 1	0.008 (-0.010, 0.026)	0.013 (-0.010, 0.035)	0.009 (-0.013, 0.030)	-0.006 (-0.027, 0.016)
	Model 2	0.011 (-0.007, 0.029)	0.014 (-0.008, 0.036)	0.012 (-0.010, 0.033)	-0.002 (-0.023, 0.020)

[a] Model was analyzed by multiple linear regression;
Model 1 was adjusted for age, gender;
Model 2 was adjusted for age, gender, living alone, educational level, marital status, physical activity, drinking, cognitive impairment, and dietary structure;
Bolding indicates *P* value < 0.05;
AA, Acetic Acid; PA, Propionic Acid; BA, Butyric Acid; VA, Valeric Acid; CA, Caproic Acid; LA, Lactic Acid; BHB, β-hydroxybutyric Acid; β, Correlation Coefficient; CI, Confidence Interval.

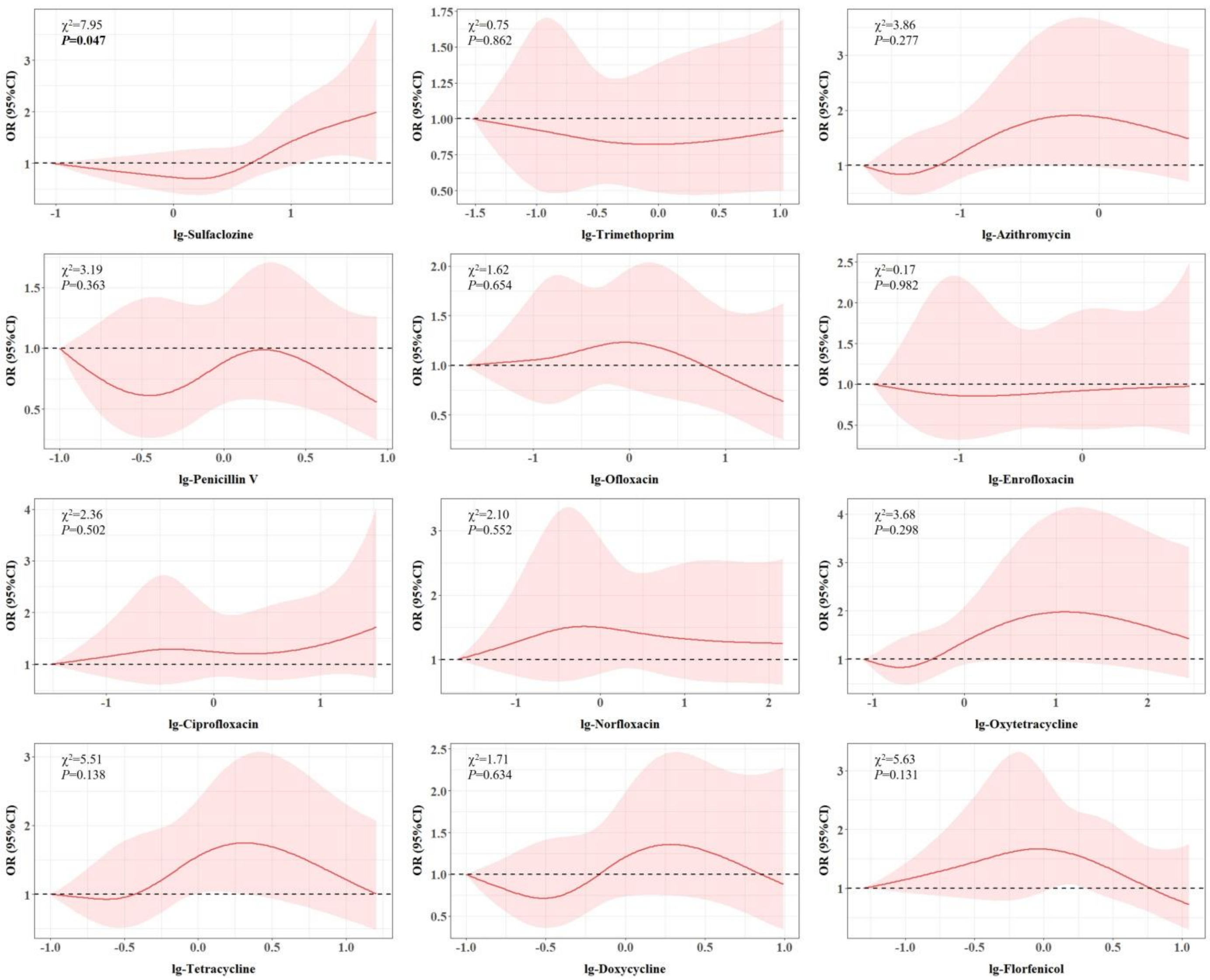

Figure S1 Nonlinear association of individual antibiotic exposure with depressive symptoms.

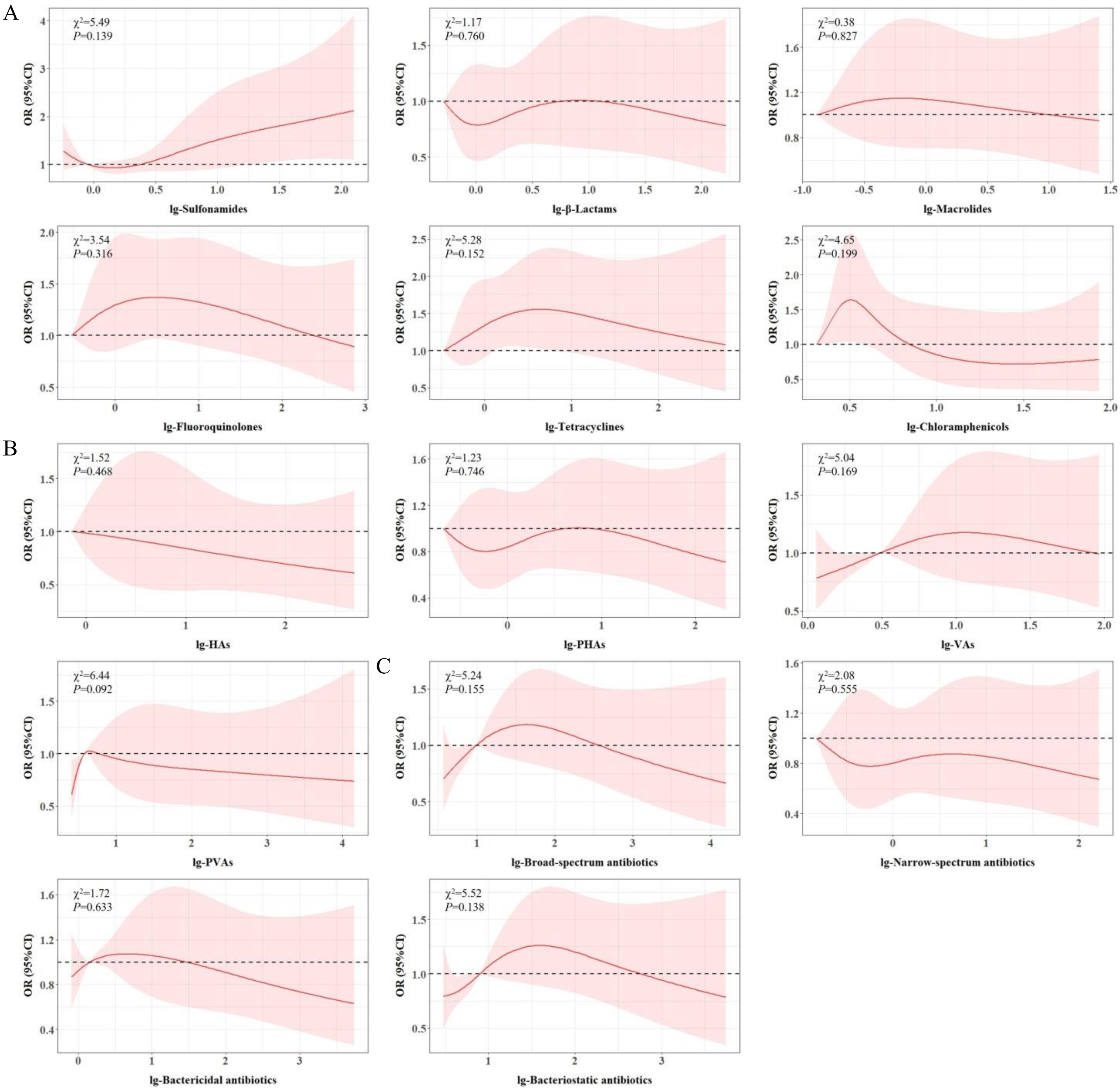

Figure S2 Nonlinear associations between exposure to different types of antibiotics and depressive symptoms. HAs, Human Antibiotics; VAs, Veterinary Antibiotics; PHAs, Antibiotics Preferred as HAs; PVAs, Antibiotics Preferred as VAs.

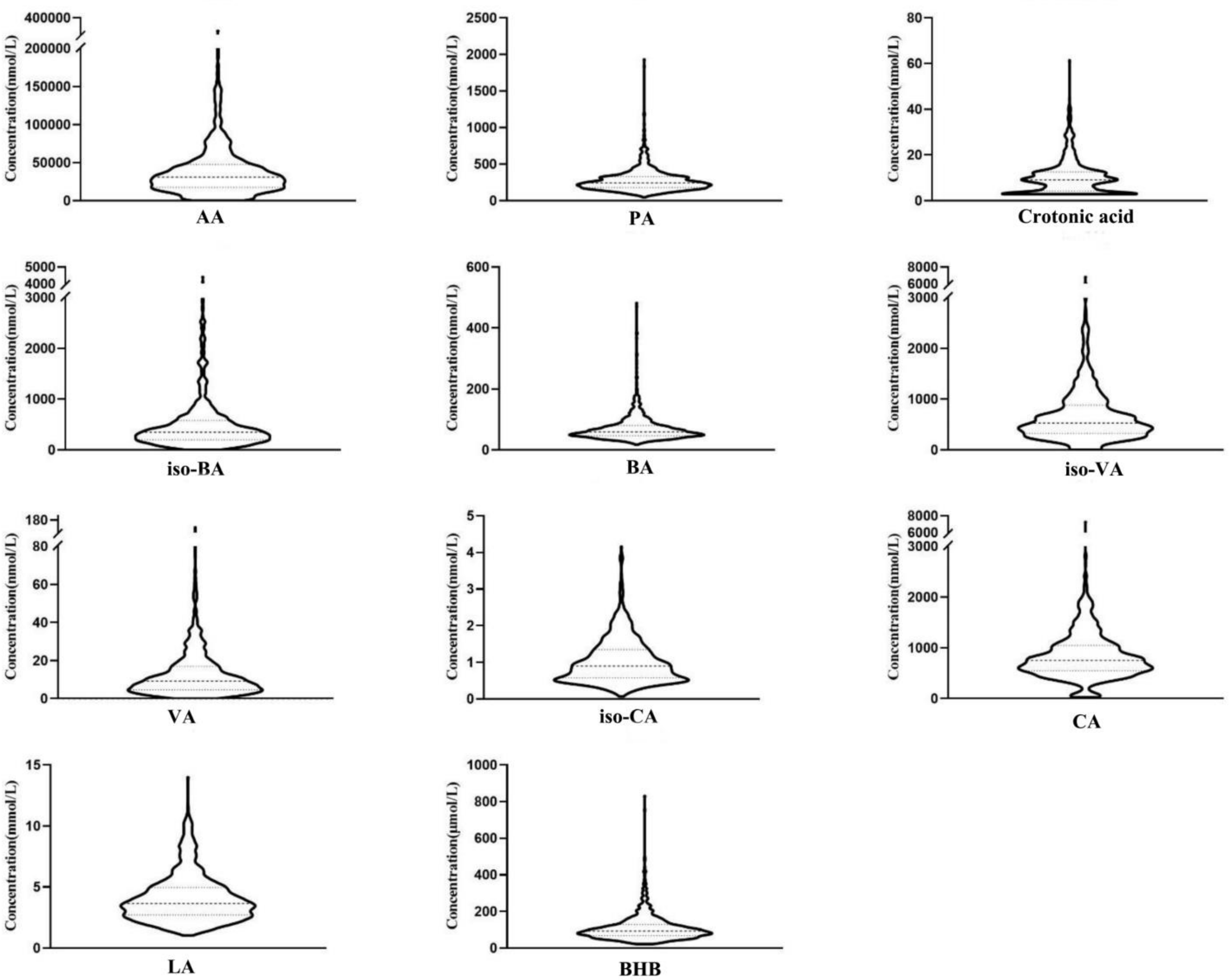

Figure S3 Distribution of short-chain organic acids in the elderly. AA, Acetic Acid; PA, Propionic Acid; BA, Butyric Acid; VA, Valeric Acid; CA, Caproic Acid; LA, Lactic Acid; BHB, β-hydroxybutyric Acid.

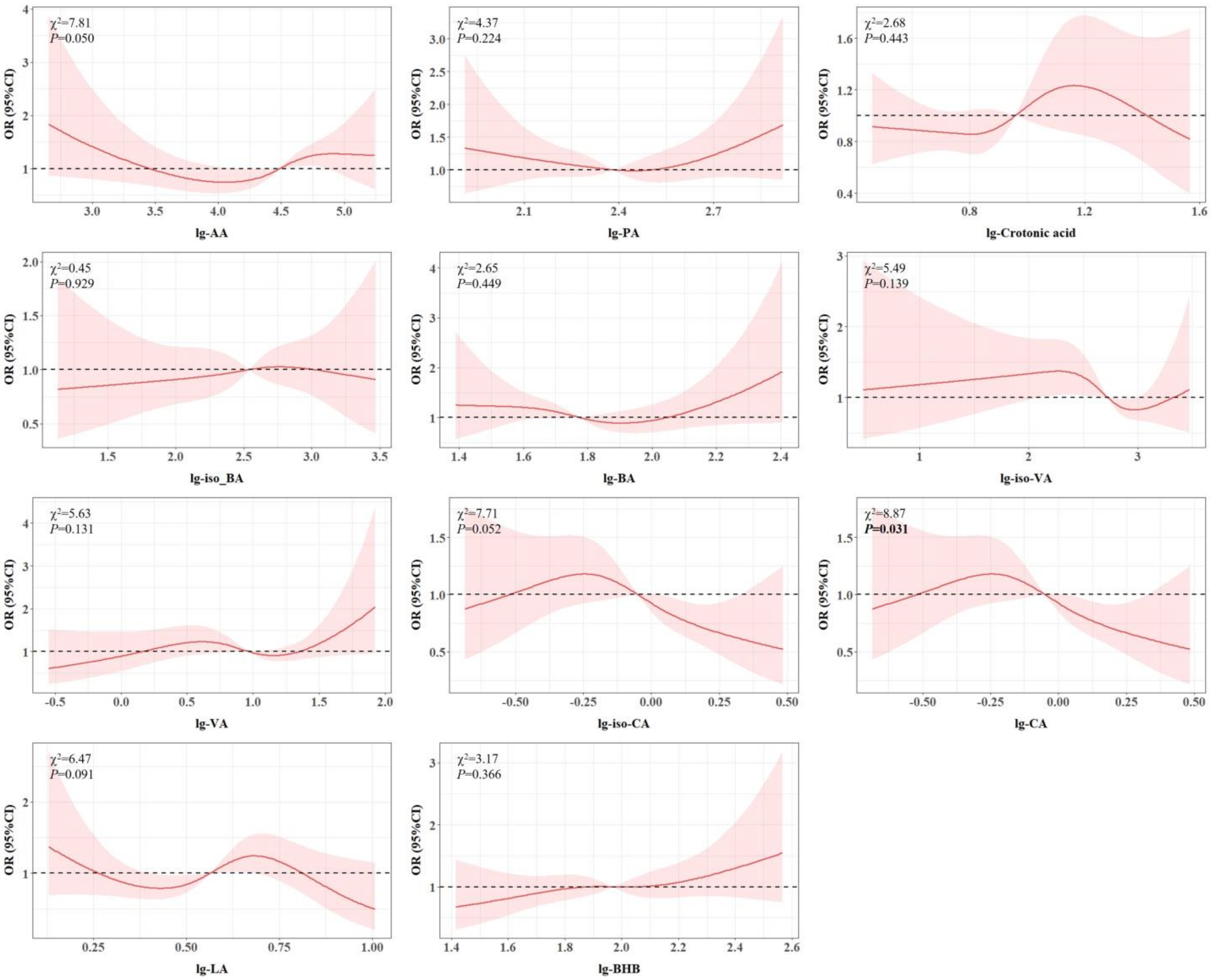

Figure S4. Nonlinear association between serum short-chain organic acids and risk of depression in the elderly. AA, Acetic Acid; PA, Propionic Acid; BA, Butyric Acid; VA, Valeric Acid; CA, Caproic Acid; LA, Lactic Acid; BHB, β-hydroxybutyric Acid.

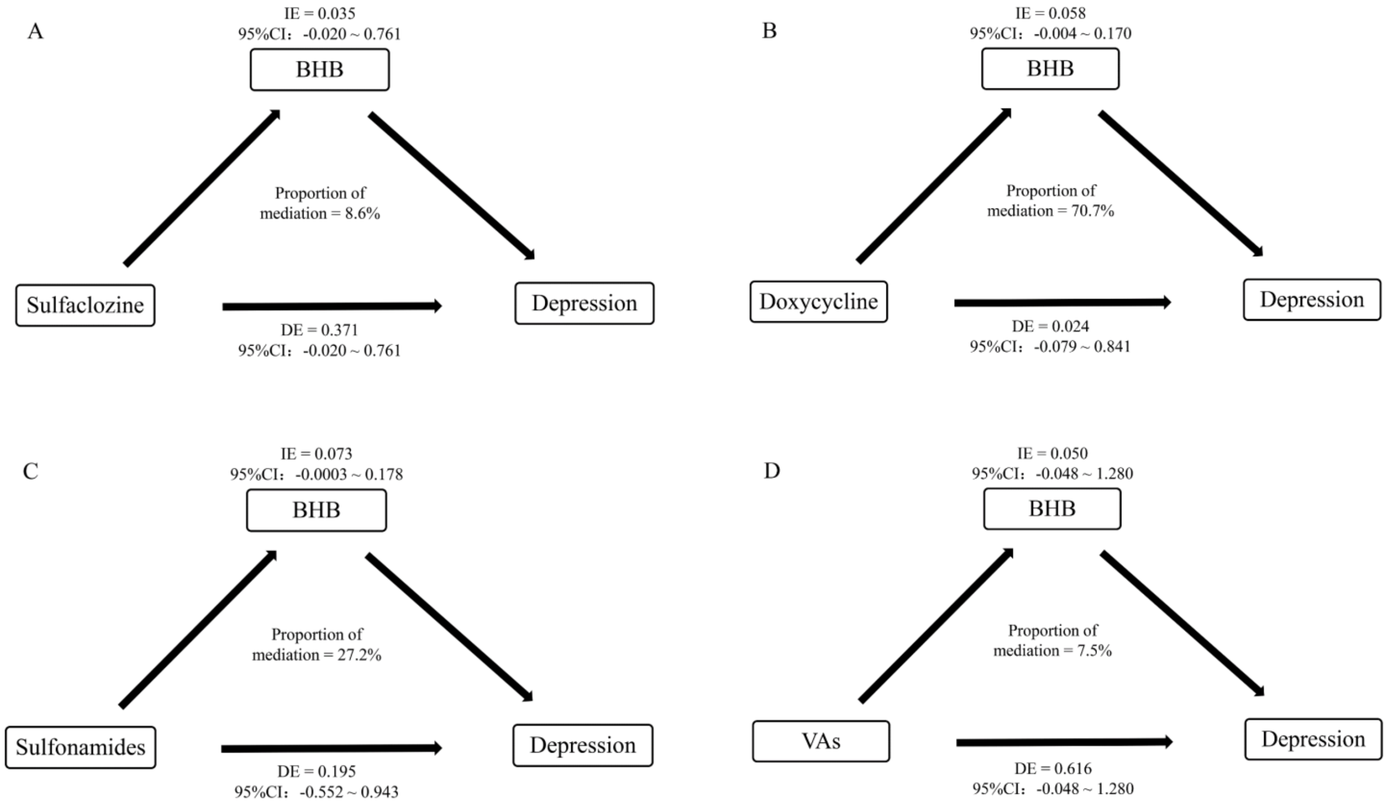

Figure S5. Intermediation of BHB. Adjusted for age, gender, living alone, educational level, marital status, physical activity, drinking, cognitive impairment, and dietary structure; BHB, β-hydroxybutyric Acid; VAs, Veterinary Antibiotics; IE, Indirect effect; DE, Direct effect; CI, Confidence Interval.
